# “Brain‐IT”: Exergame training with biofeedback breathing in neurocognitive disorders

**DOI:** 10.1002/alz.13913

**Published:** 2024-05-29

**Authors:** Patrick Manser, Eling D. de Bruin

**Affiliations:** ^1^ Motor Control and Learning Group, Institute of Human Movement Sciences and Sport, Department of Health Sciences and Technology ETH Zurich Zurich Switzerland; ^2^ Department of Health OST ‐ Eastern Swiss University of Applied Sciences St. Gallen Switzerland; ^3^ Division of Physiotherapy, Department of Neurobiology, Care Sciences, and Society Karolinska Institute Huddinge Sweden

**Keywords:** cognition, cognitive impairment, effectiveness, eHealth, exercise, exergame, neuroplasticity, neurosciences, technology, training

## Abstract

**INTRODUCTION:**

The combination of exergame‐based motor‐cognitive training with resonance breathing guided by heart‐rate variability biofeedback (HRV‐BF) targets various relevant mechanisms of action to alleviate the pathological state in mild neurocognitive disorders (mNCD).

**METHODS:**

This randomized controlled trial (RCT) investigated the effectiveness of adding this novel intervention approach to usual care in mNCD. The individualized intervention was delivered via the “Brain‐IT” training concept, which was iteratively co‐designed, tested, and refined with patient and public involvement.

**RESULTS:**

We observed statistically significant effects with large effect sizes for global cognitive performance, immediate verbal recall, and delayed verbal recall in favor of the intervention group. Fifty‐five percent of participants showed a clinically relevant improvement in response to training.

**DISCUSSION:**

Confirmatory RCTs are warranted to investigate whether the observed improvements in cognitive performance translate to affecting the rates of progression to or onset of dementia and test the implementation of the training in clinical practice.

**Highlights:**

- We proposed a novel intervention approach for mild neurocognitive disorders.
- It combines exergame‐based training with biofeedback‐guided resonance breathing.
- Our results confirm the effectiveness of this approach.
- Fifty‐five percent of participants showed a clinically relevant improvement in response to training.

## 1 BACKGROUND

The global prevalence of dementia is projected to increase dramatically, making it a key challenge for aging societies. To mitigate this impending escalation, it is imperative to implement sustainable and effective measures aimed at averting its progression.^1^ Individuals at an early stage of the disorder (mild cognitive impairment [MCI] or mild neurocognitive disorder [mNCD]^2^) may represent an optimal target population for secondary prevention.^3^

Where physical frailty can be seen as emerging from dysregulation of multiple interconnected physiological and biological systems that cross a threshold to critical dysfunction, thus severely compromising homeostasis,^4^ a similar phenomenon can be assumed for cognitive frailty. Several studies have reported the interactions between neuro‐immune, immune‐metabolic, and neuro‐metabolic pathways,^5^ which also bears relevance for individuals with Alzheimer's disease (AD).^6^, ^7^ Consequently, holistic interventions that have multisystem effects are more promising to remedy cognitive impairment than interventions targeted at replenishing single systems.

Combined physical and cognitive training was recently recommended for the secondary prevention of mNCD by a collaborative international guideline.^8^ Physical exercise is proposed to alleviate the pathological state of mNCD, which is characterized by an abnormal accumulation of proteins, excessive oxidative stress, metabolic disorder, and neuroinflammation within the brain, via distinct mechanisms of action^9^, ^10^, ^11^ that also lead to an improvement of brain structure and function^9^, ^10^, ^12^, ^13^, ^14^ and help maintain or increase cognitive reserve.^10^, ^15^ Cognitive exercises support and stabilize these neuroplastic processes, facilitating the survival and integration of new neuronal structures in brain circuits.^12^, ^13^, ^14^ The simultaneous execution of physical and cognitive exercises has positive synergistic effects and is currently considered the most effective training type for improving cognitive performance in mNCD.^11^, ^12^, ^13^, ^14^

For individuals with mNCD specifically, it is imperative to also consider that these individuals often have disrupted self‐regulatory capacity to flexibly adapt to daily life challenges.^16^ This capacity is supported by the central autonomic networks, which can be viewed as an integrated component of an internal regulatory system in which the brain controls visceromotor, neuroendocrine, and behavioral responses that are critical for goal‐directed behavior, adaptability, and health.^17^ Therefore, interventions should be designed holistically to also target this network specifically.

This could be achieved by combining simultaneous motor‐cognitive training with resonance breathing guided by heart‐rate variability biofeedback (HRV‐BF). HRV‐BF entails procedures that consist of feeding back beat‐by‐beat HRV data during slow breathing maneuvers so that the participant attempts to maximize certain variables of HRV, for example, to create a sine‐wave–like curve of peaks and valleys, and to match respiratory sinus arrhythmia to heart‐rate patterns.^18^ HRV‐BF aims to increase cardiac autonomic control, enhance homeostatic regulation, and regulate emotional state.^18^, ^19^, ^20^ An increased cardiac autonomic control increases vagal afferent transmission to the forebrain and activates and stimulates brain regions relevant to cognitive adaptations (such as the prefrontal cortex).^19^, ^20^ HRV‐BF or paced breathing (at resonance frequency) is effective in improving cardiac autonomic control,^20^, ^21^ cognitive functioning (in particular executive functions),^22^, ^23^ and emotional regulation^20^, ^23^ (i.e., by decreasing symptoms of depression,^20^, ^23^, ^24^ anxiety,^20^, ^24^, ^25^ and stress^24^, ^25^) across different age groups and in clinical populations. Moreover, there is evidence supporting a causal role of cardiac autonomic control in modulating plasma AD‐related biomarkers.^26^

**RESEARCH IN CONTEXT**

1. **Systematic review**: The authors reviewed the scientific literature and proposed a novel intervention approach of combining exergame‐based motor‐cognitive training with resonance breathing guided by heart‐rate variability biofeedback to optimally target relevant mechanisms of action to alleviate the pathological state of mild neurocognitive disorder (mNCD). An individualized training concept implementing this novel intervention approach was then iteratively co‐designed, tested, and refined until a safe, feasible, usable, and well‐accepted solution was achieved.
2. **Interpretation**: This study's results offer robust evidence that the resulting training is effective and thus represents a well‐accepted and scalable non‐pharmacological adjunctive therapy to standard treatment. Additionally, it was hypothesized that this new intervention approach may have positive synergistic effects.
3. **Future directions**: Confirmatory randomized controlled trials are necessary to evaluate the intervention's long‐term (transfer) effects, elucidate its biological mechanisms of action, test the new hypothesis of positive synergistic effects, and investigate the implementation of the training in clinical practice.

Although HRV‐BF has been suggested to be useful as a complementary treatment,^23^ its combination with simultaneous motor–cognitive training remains to be investigated. In light of a holistic approach that maximizes transferability to clinical practice, this new intervention approach should be implemented using technological innovations, such as exergames.^27^ Exergames offer a standardized and scalable method for training and can be designed to provide an optimal environment with enriched multisensory feedback to enhance skill acquisition and neuroplasticity^28^ in a motivating environment^29^ that promotes positive behavioral changes^30^ and typically resulting in high training adherence.^29^ As a result, exergames are currently considered more promising than conventional training approaches.^31^, ^32^, ^33^

This is the first randomized controlled trial (RCT) aiming to investigate the effectiveness of the combination of exergame‐based motor‐cognitive training with HRV‐BF delivered via an individualized exergame‐based training concept (called “Brain‐IT”) in individuals with mNCD.

## 2 METHODS

### 2.1 Prior work

In the project “Brain‐IT,” we systematically designed and developed a novel training concept (“Brain‐IT”) specifically for older adults with mNCD that addresses the proposed mechanism of action described in the introduction.

The project's methodology^34^ followed the guidelines of the Medical Research Council for the development and evaluation of complex interventions^35^ as well as the Multidisciplinary Iterative Design of Exergames (MIDE) framework.^36^ The “Brain‐IT” project was structured in three phases. In phase 1, we systematically combined a comprehensive literature synthesis^34^ with qualitative research including primary end users (older adults with mNCD), secondary end users (physiotherapists, occupational therapists, health‐care professionals), exergaming researchers, as well as experts from the exergaming industry^37^ to specify a set of design requirements for the “Brain‐IT” training concept. In phase 2, possible concepts for the exergame‐based training concept were co‐designed and elaborated based on the set of design requirements defined in phase 1. The first prototype of the resulting “Brain‐IT” training concept^34^ then entered the iterative cycle of feasibility, usability, safety and acceptance testing, and integrating study results for further development based on co‐design until an “acceptable” solution was achieved. The results of this process revealed that the resulting “Brain‐IT” training is feasible, usable, safe, and highly accepted by older adults with mNCD and preliminary data on the effects of the “Brain‐IT” training are promising.^38^ This study is part of phase 3 of the project.

### 2.2 Objectives and hypotheses

This study evaluated the effectiveness of adding “Brain‐IT” training to usual care in improving global cognitive performance in older adults with mNCD compared to usual care alone. As secondary objectives, the effects of the “Brain‐IT” training on: (1) domain‐specific cognitive performance (i.e., learning and memory, complex attention, executive function, and visuospatial skills), (2) spatiotemporal parameters of gait, (3) instrumental activities of daily living (IADL), and (4) psychosocial factors (i.e., quality of life [QoL], and levels of depression, anxiety, stress), and (5) cardiac vagal modulation (i.e., resting vagally mediated heart‐rate variability [vm‐HRV]) in older adults with mNCD compared to usual care were explored. The specific hypotheses were detailed in the published study protocol.^39^ Additionally, the study protocol states that we aimed to evaluate brain structure and function to explore possible underlying neural changes of the training in relation to adaptations in cognitive performance. Because details on the methods for these analyses are dependent on the results on cognitive performance reported here, these will be reported separately in focused manuscripts.

### 2.3 Explanation and choice of comparators

As detailed in the study protocol,^39^ we have chosen to compare the addition of the “Brain‐IT” training to usual care versus usual care alone as a comparator. This decision is based on the alignment of recommended treatment and management of individuals with mNCD in Switzerland with available global clinical practice guidelines and consensus statements.^40^

### 2.4 Protocol and registration

The study protocol for this RCT was prepared in accordance with established guidelines from the “SPIRIT 2013 Statement: Defining Standard Protocol Items for Clinical Trials”^41^, ^42^ and published previously.^39^ In this article, key information is reported to adhere to the latest version of the “Consolidated Standards of Reporting Trials (CONSORT) Statement for Randomized Trials of Nonpharmacologic Treatments”^43^ (checklist see Supplementary File 1 of File S1 in supporting information). For full reproducibility, please also refer to the study protocol.^39^

#### 2.4.1 Important changes to the trial design and study setting after commencement

Recruitment was extended by 6 weeks. This allowed us to stop recruitment only once we had complete data on the primary outcome for the planned minimum sample size of 34 participants. Other than that, there were no changes to, or deviations from, the published study protocol.

### 2.5 Overview of the trial design, participants, and interventions

A two‐arm, prospective, parallel‐group, single‐blinded (i.e., outcome evaluator of pre‐ and postmeasurements blinded to group allocation) RCT with a 1:1 allocation ratio (i.e., intervention:control) including older adults with mNCD was conducted between May 2022 and February 2024. The study was registered at ClinicalTrials.gov prior to the start of patients’ recruitment (NCT05387057; date of registration: May 18, 2022) The study set‐up was multicentric (Zurich and St. Gallen) and national (Switzerland).

Individuals with mNCD were recruited between May 2022 and October 2023 in collaboration with (memory) clinics in the larger area of Zurich and St. Gallen. Suitable patients were either identified from medical records and patient registries of (memory) clinics or from recent clinical diagnostics performed by their medical doctors or therapists authorized to search medical records. Alternatively, suitable patients were identified by an informant (i.e., health‐care professionals)‐based suspicion of MCI of one of their patients. To ensure diversity, equity, and inclusion, all patients referred to us by the clinical recruitment partners were fully considered for participation in the study. After recruitment and providing written informed consent, participants were screened on eligibility (for a full list of eligibility criteria see study protocol^39^), and premeasurements were scheduled for all eligible participants.

Pre‐ and postmeasurements took place at one of our study sites (ETH Hönggerberg, Zurich and Eastern Switzerland University of Applied Sciences, St. Gallen) within 2 weeks prior to starting and after completing the intervention. The measurements took approximately 90 minutes. All participants having no contraindications to magnetic resonance imaging (MRI) had an additional appointment to conduct an MRI scan (duration: approximately 1 hour [including preparation]) at University Hospital Zurich, which included an isotropic t_1_‐weighted scan, diffusion tensor imaging, as well as resting and task‐based functional MRI. All measurements were led by two investigators of our research team trained in the application of the measurement techniques and protocols. Pre‐ and postmeasurements were scheduled to take place at approximately the same time of the day (±2 hours) for each participant. To minimize the influence of transient confounding effects on vm‐HRV, all participants were additionally instructed verbally and in writing to follow a normal sleep routine the day before the experiment, to avoid intense physical activities and alcohol consumption within 24 hours before measurements, and to refrain from coffee or caffeinated drinks as well as food consumption at least 2 hours before measurements.^44^

After completing premeasurements, participants were randomly allocated to the intervention or control group and were instructed about their respective intervention procedures. The control group proceeded with usual care as provided by the (memory) clinics where the patients were recruited. Usual care for mNCD typically includes medication (e.g., cholinesterase inhibitors), treating medical conditions other than mNCD (e.g., diabetes mellitus and depressive symptoms), controlling comorbidities (e.g., hypertension and obesity), and managing risk factors (e.g., smoking habits and physical and cognitive inactivity), as well as recommendations for changing lifestyle habits (e.g., living a cognitively, physically, and socially active life), physiotherapy to treat specific health problems, such as back pain or mobility problems, occupational therapy, or day clinic visits. For participants in the intervention group, the exergame device was installed at their homes, they got safety instructions, and were familiar with the exergame training system and then started with their 12‐week training intervention according to the “Brain‐IT” training concept in addition to usual care. The “Brain‐IT” training concept represents a guideline for applying a combination of exergame‐based motor‐cognitive training and HRV‐BF training by standardizing the training characteristics (e.g., training frequency, intensity, and duration), as well as the structure and content of training, whereas the exergame device and the specific games used within each of the defined neurocognitive domains can be replaced by alternative exergames. To ensure replicability, the “Brain‐IT” training concept was planned and reported according to the Consensus on Exercise Reporting Template (CERT)^45^ and provides specific instructions on how to adapt the “Brain‐IT” training concept to other hardware and software solutions (see Supplementary File 2 of File S2 in supporting information).

For an overview, the “Brain‐IT” training consists of a personalized and individually adapted multidomain exergame‐based simultaneous motor‐cognitive training with incorporated cognitive tasks (i.e., as defined by Herold et al.,^14^ with the exception of the walking on the spot task in the “Phase 1 ‐ Facilitation” of the training structure to ensure the moderate physical exercise intensity, which is an additional motor training element that is added to the remaining simultaneous‐integrated motor‐cognitive tasks) combined with HRV‐BF training. It is adopted with a deficit‐oriented focus on the neurocognitive domains of (1) learning and memory, (2) executive function, (3) complex attention, and (4) visuospatial skills. Each participant was instructed to train ≥ 5×/week for ≥ 24 minutes per session resulting in a weekly training volume of ≥ 120 minutes. All training sessions took place at participants' homes. As per the original “Brain‐IT” training concept,^34^ 19 to 24 training sessions were supervised by a designated investigator who instructed and oversaw the participants' use of the exergame device, ensured safety protocols were followed (e.g., ensuring that there were no hard objects [e.g., couch table] within the potential drop zone, determining the appropriate level of stability support using walking sticks, handrail, or similar), and ensured adherence to the “Brain‐IT” training concept. All deviations from the “Brain‐IT” training concept were reported. In this project, we used technology from Dividat AG (i.e., “Senso Flex” [Dividat AG, Schindellegi, Switzerland; hardware: prototype version 2, software: version 22.4.0‐360‐gf9df00d5b], Polar [i.e., heart‐rate monitor (Polar M430) and sensor (Polar H10)], and Kubios [Kubios HRV Premium (Kubios Oy, Kuopio, Finland, version 3.4)]) to implement our training concept. For more detail on how the specific technologies are used to implement our training concept, consider the revised version of the “Brain‐IT” training concept evaluated in this study (Supplementary File 2 of File S2 in supporting information).

After completing the 12‐week intervention period, postmeasurements were performed for both groups. An individual report of their results was provided to each participant and discussed with them personally. In addition, viable options for continuing (or, for the control group, starting) “Brain‐IT” training outside of the study were carefully explored and identified, and we provided support for their implementation. No compensation was granted to participants, but detailed feedback on individual performance as well as the study outcomes in general was provided at the end of the trial. When possible, caregivers were actively involved in helping participants travel to the study centers for measurements and reminding them to adhere to the training plan.

For readers interested in a visual insight into the study, a promotional video of this RCT is available on our project homepage^46^ (i.e., Promo Video 2.0; published March 7, 2023).

### 2.6 Overview of outcomes

An overview of all outcome measures is provided below. Details for all specific assessments and measurement conditions are provided in the published study protocol. Due to the journal's stipulations on the maximum number of references, all references to the respective assessments are also provided in the published study protocol.^39^

#### 2.6.1 Primary outcome

As primary outcome, changes in global cognitive performance were assessed using the validated German version^47^ of the Quick Mild Cognitive Impairment screen (QMCI).^48^, ^49^ The QMCI comprises six subtests: orientation (10 points), registration (5 points), clock drawing (15 points), delayed recall (20 points), verbal fluency (20 points), and logical memory (30 points); it was scored as a point rate out of a maximum score of 100^49^ and was shown to be sensitive for changes in cognitive performance over time.^50^ The QMCI was administered and evaluated according to published guidelines.^49^ A clinically meaningful change was defined as a change in ≥ 3 points in the QMCI score.^39^

#### 2.6.2 Secondary outcomes

As secondary outcomes, key neurocognitive domains (as defined in Sachdev et al.,^51^ in line with the Diagnostic and Statistical Manual of Mental Disorders 5th Edition [DSM‐5]^52^ and according to recommendations^3^, ^53^) of (1) learning and memory; (2) complex attention; (3) executive function; and (4) visuospatial skills, spatiotemporal parameters of gait, psychosocial factors (i.e., QoL, and levels of depression, anxiety, stress), and cardiac vagal modulation (resting vm‐HRV) were assessed.

As defined in the published study protocol,^39^ learning and memory was assessed using the German version of the subtests “logical memory” of the Wechsler Memory Scale ‐ fourth edition (WMS‐IV‐LM) and a computerized version of the Digit Span Forward test (Psychology Experiment Building Language [PEBL]‐Digit Span Forward [PEBL‐DSF]). Complex attention was assessed using a computerized version of the Trail Making Test, Part A (PEBL‐TMT‐A) and the subtest “Go‐NoGo” of the Test of Attentional Performance (TAP Go‐NoGo). Executive function was assessed considering planning abilities (i.e., using the HOTAP picture‐sorting test part A [HOTAP‐A]), working memory (i.e., using a computerized version of the Digit Span Backward test [PEBL Digit Span Backward (PEBL‐DSB)]), and cognitive flexibility (i.e., using a computerized version of the Trail Making Test, Part B [PEBL‐TMT‐B]). Visuo‐spatial skills were tested with a computerized version of the classic Shepard and Metzler mental rotation task, that was executed using PEBL Test battery software (version 2.1 [2]; with default settings). Spatiotemporal gait parameters were assessed using a BTS G‐WALK® (BTS Bioengineering SpA) inertial sensor gait‐analysis protocol consisting of a figure‐8 walking path (i.e., distance between cones approximately 8 meters). IADL functioning was assessed by report of the closest informant (e.g., spouse, child, or friend) using the Amsterdam IADL Questionnaire short version German for Switzerland. QoL was evaluated in interview format using the validated German version of the Quality of Life‐AD (QoL‐AD) scale. Levels of depression, anxiety, and stress were assessed using the validated German version of the Depression Anxiety Stress Scale‐21 (DASS‐21). Resting vm‐HRV was assessed in accordance with recommendations for experiment planning, data analysis, and data reporting using a heart‐rate monitor (Polar M430) and sensor (Polar H10). Data were analyzed in Kubios HRV Premium (Kubios Oy, version 3.4) using the validated beat correction algorithm and noise handling provided by the software.

#### 2.6.3 Other endpoints

We kept a protocol of all (serious) adverse events ([S]AEs).

Baseline factors were collected through demographic data including age, sex, height, weight, body mass index (BMI), years of education, physical activity behavior (i.e., measured with the German version of the International Physical Activity Questionnaire Short Form ‐ short form [IPAQ‐SF]^39^), etiological subtype (i.e., mNCD due to AD, mild frontotemporal NCD, mNCD with Lewy bodies, or mild vascular NCD).

In Switzerland, usual care is highly individual, varies between (memory) clinics where patients are recruited, and it is unclear whether patients comply with the recommendations of their clinicians. Therefore, details about all structured or guided usual care activities or both, medication intake at baseline, as well as changes in medication intake between pre‐ and postmeasurements were assessed in both the intervention and control groups.

Actual delivery of the “Brain‐IT” training was measured with an attendance adherence (number of sessions completed per week per participant) and duration adherence (training time completed per week per participant) protocol, which was automatically assessed in the exergame training software. To ensure that participants who trained more than the prescribed minimum frequency did not compensate for lower adherence rates in other participants or training weeks in which they trained less, mean adherence rates were calculated as the average of each participant's weekly attendance and duration adherence with a maximum of 100% (for formulae for calculations see Manser et al.^38^). Reasons for non‐adherence and dropouts were recorded.

### 2.7 Sample size

The sample size was justified based on Whitehead et al.^54^ and the following assumptions: the future main (full‐scale) RCT is planned with identical design and primary outcome as this study, with a two‐sided type 1 error rate of 5% and a statistical power of 80%. A medium effect size (i.e., standardized mean difference of 0.5 based on previous meta‐analytic findings) and an attrition rate of 20% were anticipated. For more details on how we derived these anticipated effect sizes and attrition rate, please refer to the study protocol.^39^ The effect estimate measure of a standardized mean difference (instead of partial eta‐squared) was used because this was required to estimate the required sample size that provides a sufficiently precise estimate of the treatment effect to minimize the sample needed for a future full‐scale RCT based on the methods of Whitehead et al.^53^ This method is designed for research stages in which no data from pilot studies or similar are available for a robust sample size calculation. To ensure an adequate number of participants, a wide upper safety margin for an attrition rate of up to 40% was chosen. Based on these considerations, we aimed to recruit *n* = 17 to 20 older adults with mNCD per group, leading to a total sample size of *N* = 34 to 40. This provides a sufficiently precise estimate of the treatment effect to minimize the sample needed for a future full‐scale RCT.^54^

### 2.8 Randomization

To ensure allocation concealment, each participant was individually assigned to intervention or control group by the investigator assigned as responsible person for supervision and correspondence with the respective study participant after completing premeasurements. A variable block randomization model (i.e., block sizes = 4, 6, 8) implemented in the data management system Castor EDC (Ciwit BV)^55^ with a 1:1 allocation ratio stratified by sex and per institute (study center) was used.

### 2.9 Blinding

Outcome assessors of the pre‐ and postmeasurements were blinded to group allocation (single blinding). To ensure blinding and blind‐keeping of all outcome assessors, detailed study‐specific guidelines for all relevant procedures have been established that were strictly followed by all involved study investigators. For data assessed throughout the intervention period (i.e., only applicable for intervention group), blinding of investigators was not possible. Blinding of participants was also not be possible because usual care was used as a control intervention.

### 2.10 Participant retention

Once a participant was included, a trained investigator was assigned as the person responsible for supervision and correspondence with the respective study participant and made all reasonable efforts to achieve the participant's retention in the study. Examples include providing written information sheets and reminders about study appointments, involving caregivers or relatives as personal support for study participants, and providing assistance with travel to the study center. Specifically, in the intervention group, each participant was provided with a detailed training manual that was individually adapted to the participant's set‐up to help them use the training system correctly (with photographs and explanations for each step from starting the system to training completion, including a colored step‐by‐step identification of required elements [cables and buttons]). Furthermore, the study team provided telephone support in the case of technical difficulties or comprehension problems for unsupervised training sessions.

### 2.11 Statistical methods

Statistical analysis was done using R (The R Foundation; version 4.3.1 GUI 1.79 Big Sur Intel build) in line with RStudio (RStudio, Inc.; version 2023.12.1+402). We did a modified intention‐to‐treat analysis (i.e., data of all randomized participants who completed pre‐ and post measurements, regardless of protocol adherence, were included in statistical analyses). Questionnaire scores were regarded as ordinary data. Data was reported as mean ± standard deviation for data fulfilling all the assumptions that would subsequently justify parametric statistical analyses. In case these assumptions were not met, medians (interquartile ranges) were reported.

For all outcomes, descriptive statistics were computed first. Normality distribution of data was checked using a Shapiro–Wilk test. Level of significance was set to *P* ≤ 0.05 (two sided, uncorrected).

For all demographic variables, between‐group differences (i.e., intervention vs. control) were tested using an independent *t* test or Mann–Whitney *U* test in case the data were not normally distributed. Between‐group differences in categorical variables were tested using the Fisher exact test. To discover whether the between‐group differences were substantive, Pearson *r* effect sizes were calculated^56^ and interpreted to be small (0.1 ≤ *r* < 0.3), medium (0.3 ≤ *r* < 0.5) or large (*r* > 0.5).^57^

For the primary and all secondary outcomes, the assumption of homogeneity of variance was checked using a Levene test. In case all assumptions for analysis of covariance (ANCOVA) were met, the effectiveness of the “Brain‐IT” training was evaluated using an ANCOVA with the pre‐measurement value as a covariate for the predicting group factor and the postmeasurement value as outcome variable.^56^ In case not all assumptions were met, a Quade non‐parametric ANCOVA was used. To discover whether effects are substantive, partial eta‐squared (η^2^ *_p_*) effect sizes for the covariate‐adjusted between‐group postmeasurement differences derived from the ANCOVA were calculated for all primary and secondary endpoints. Effect sizes were interpreted to be small (0.01 ≤ η^2^ *_p_* < 0.06), medium (0.06 ≤ η^2^ *_p_* < 0.14) or large (η^2^ *_p_* ≥ 0.14).^57^

Statistical analysis was done after data collection was completed. No interim, subgroup, or adjusted analyses were performed.

## 3 RESULTS

### 3.1 Recruitment and participant flow

**FIGURE 1:**
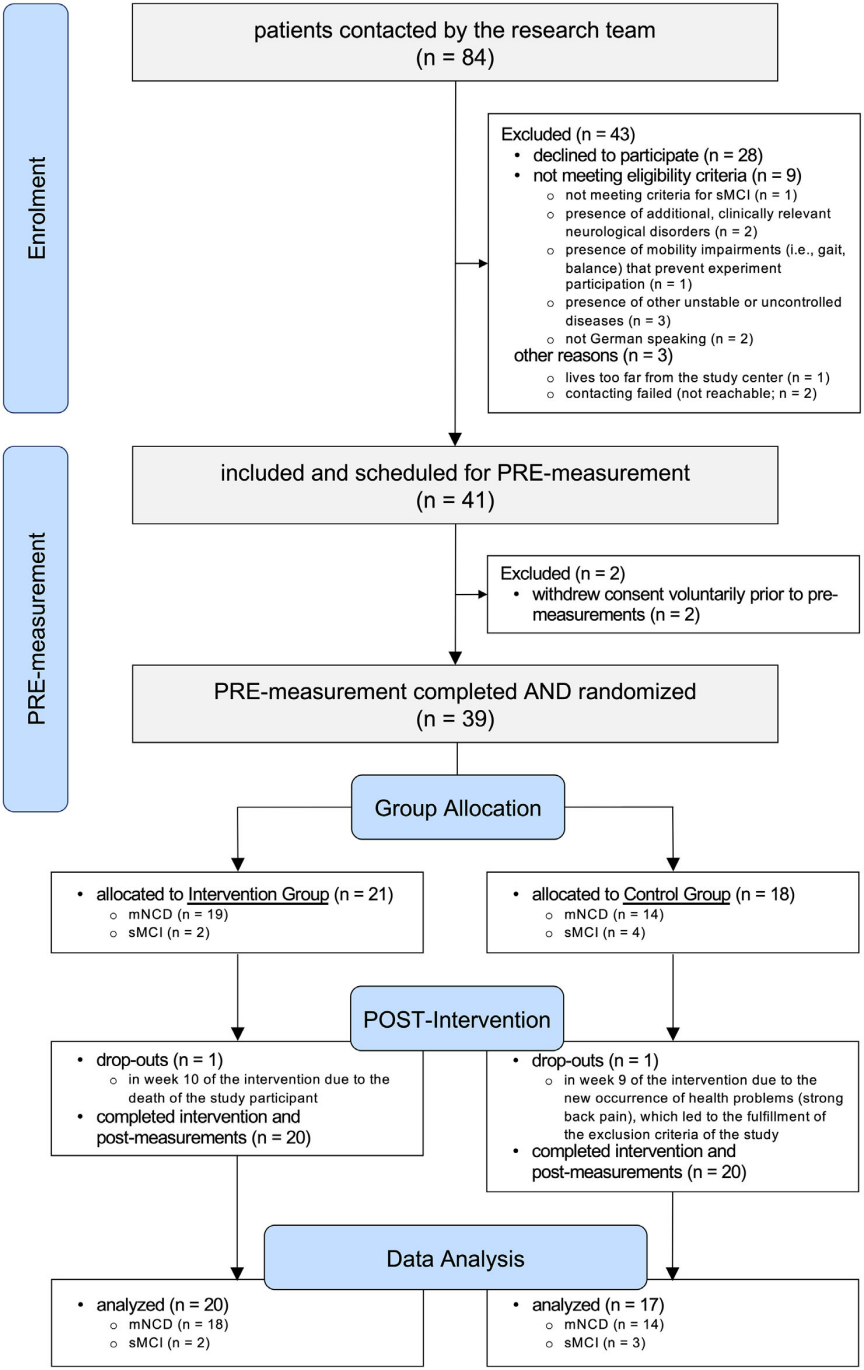
Summary of the participants’ flow throughout the study. mNCD, clinically diagnosed mild neurocognitive disorder; sMCI, screened for mild cognitive impairment

A summary of the flow of participants through the study is shown in Figure 1. Recruitment was stopped when we had complete data on the primary outcome for the planned minimum sample size of 34 participants. A total of 41 participants were enrolled, of whom two withdrew consent voluntarily prior to premeasurements and two dropped out during the intervention (one in each group). As a result, 37 participants (72.8 ± 9.0 years; 30% females) successfully completed the study. Of these 37 participants, 32 were clinically diagnosed with mNCD and five met the criteria defined for patients positively screened for MCI. No deviations from the study protocol regarding initiation of the interventions (within 2 weeks after completing premeasurements) were recorded. No intervention‐related (S)AEs were recorded.

### 3.2 Baseline data

Table 1 summarizes the demographic characteristics of the participants. A moderate effect size was observed for a higher BMI in the intervention group, although the mean/median of both groups fell within the range of a “healthy” BMI. No other statistically significant between‐group differences were found.

**TABLE 1:** Demographic characteristics of the study population.

			Between‐group difference		
	Group: “Brain‐IT” training (*n* = 20)	Group: usual care (*n* = 17)	Test statistics^a^	*P* value^b^	|Effect size|^c^
Age [years]	74.0 ± 8.0	71.8 ± 9.9	t = 1.048	0.304	*r* = 0.192
Sex [% females]	30	29	N/A	1.00	OR = 1.03
Body mass index [kg·m^−2^]	24.7 ± 2.3	22.7 (2.4)	W = 249	0.017*	*r*_s_ = 0.393
Years of education [years]	16.0 ± 3.5	15.5 ± 3.5	t =−0.025	0.981	*r* = 0.004
IPAQ‐SF [MET·week^−1^]	1683 (1735)	1485 (1342)	W = 219	0.141	*r*_s_ = 0.242
**Etiology of mNCD**:					
mNCD due to Alzheimer's disease	*n* = 11 (55%)	*n* = 10 (59%)	N/A	1.00	OR = 0.86
Mild frontotemporal NCD	*n* = 3 (15%)	*n* = 0 (0%)	N/A	0.234	OR = ∞
Mild vascular NCD	*n* = 3 (15%)	*n* = 5 (29%)	N/A	0.428	OR = 0.43
mNCD with Lewy bodies	*n* = 0 (0%)	*n* = 1 (6%)	N/A	0.460	OR = 0.00
Unclear/not yet determined	*n* = 3 (15%)	*n* = 1 (6%)	N/A	0.609	OR = 2.75

*Notes*: Data are reported as mean ± standard deviation for parametric analyses and median (interquartile range) for non‐parametric analyses.

Abbreviations: IPAQ‐SF, International Physical Activity Questionnaire Short Form; IQR, interquartile rage; MET, metabolic equivalent of task; mNCD, mild neurocognitive disorder; OR, odds ratio; SD, standard deviation.

^a^ *t* statistics for the between‐group differences tested with an independent *t* test or Mann–Whitney *U* test in case the data are not normally distributed.

^b^ *P* values for the between‐group differences tested with an independent *t* test or Mann–Whitney *U* test in case the data are not normally distributed, or Fisher exact test for categorical variables.

^c^ Effect size estimates for the between‐group differences tested with an independent *t* test (effect size Pearson *r*) or Mann–Whitney *U* test (effect size Spearman rho [*r*_s_]) in case the data are not normally distributed, or Fisher exact test for categorical variables (odds ratio).

* = statistically significant at *P* < 0.05.

### 3.3 Delivery of the interventions

#### 3.3.1 Type of usual care activities

For participants who completed the study, 100% of participants in the intervention group and 94% of participants in the control group reported that they received one or more structured or guided usual care activities(s) during study participation. Details on types of usual care activities are summarized in Table 2. There were no statistically significant between‐group differences in any of the pharmacological and non‐pharmacological interventions. In addition to the interventions listed in the table, one participant in the intervention group underwent surgery for an inguinal hernia and received general anesthesia 9 weeks before the postmeasurement. However, the participant was able to resume training after a brief break. In addition, one participant from the control group acquired the “Senso Flex” device from Dividat AG without informing us, as they were frustrated with the group assignment, and trained with the commercially available software of the device using individualized progression algorithms for 15 minutes per day for about 2.5 months prior to the postmeasurements.

**TABLE 2:** Type of usual care interventions.

	Proportion of participants having received the respective intervention during study participation		Between‐group difference
Type of usual care activities	Group: "Brain‐IT" training (*n* = 20)	Group: usual care (*n* = 17)	*P* value^d^ and OR
**Regular medication intake (at baseline)**:	*n* = 20 (100% of participants)	*n* = 15 (88% of participants)	*P* = 0.204, OR = ∞
**Alzheimer's disease medication**:			
Cholinesterase inhibitors	*n* = 3 (15% of participants)	*n* = 7 (41% of participants)	*P* = 0.136, OR = 0.26
**Blood pressure regulators**:			
Angiotensin‐converting enzyme inhibitors	*n* = 10 (50% of participants)	*n* = 7 (41% of participants)	*P* = 0.743, OR = 1.41
Beta‐blockers	*n* = 3 (15% of participants)	*n* = 2 (12% of participants)	*P* = 1.00, OR = 1.31
Calcium channel blockers	*n* = 4 (20% of participants)	*n* = 1 (6% of participants)	*P* = 0.348, OR = 3.87
Others	*n* = 1 (5% of participants)	*n* = 1 (6% of participants)	*P* = 1.00, OR = 0.85
**Cholesterol‐lowering agents**:			
Cholesterol absorption inhibitor	*n* = 1 (5% of participants)	*n* = 1 (6% of participants)	*P* = 1.00, OR = 0.85
Statins	*n* = 9 (45% of participants)	*n* = 6 (35% of participants)	*P* = 0.738, OR = 1.48
**Anticoagulants and antiplatelet agents**:			
Anticoagulants	*n* = 3 (15% of participants)	*n* = 3 (18% of participants)	*P* = 1.00, OR = 0.83
Antiplatelet agents	*n* = 8 (40% of participants)	*n* = 4 (24% of participants)	*P* = 0.319, OR = 2.12
**Psychopharmaka**:			
Antidepressants	*n* = 4 (20% of participants)	*n* = 3 (18% of participants)	*P* = 1.00, OR = 1.16
Antipsychotics	*n* = 1 (5% of participants)	*n* = 0 (0% of participants)	*P* = 1.00, OR = ∞
**Antidiabetic agents**:			
Glucagon‐like peptide‐1 receptor agonist	*n* = 1 (5% of participants)	*n* = 0 (0% of participants)	*P* = 1.00, OR = ∞
Insulin	*n* = 2 (10% of participants)	*n* = 1 (6% of participants)	*P* = 1.00, OR = 1.75
Metformin	*n* = 1 (5% of participants)	*n* = 2 (12% of participants)	*P* = 0.584, OR = 0.40
Sodium‐glucose transport protein 2 inhibitors	*n* = 0 (0% of participants)	*n* = 1 (6% of participants)	*P* = 0.460, OR = 0
Sulfonylurea antidiabetic agent	*n* = 2 (10% of participants)	*n* = 1 (6% of participants)	*P* = 1.00, OR = 1.75
**Other medications**:			
Betahistini dihydrochloridumistamine	*n* = 1 (5% of participants)	*n* = 0 (0% of participants)	*P* = 1.00, OR = ∞
Desmopressin	*n* = 1 (5% of participants)	*n* = 0 (0% of participants)	*P* = 1.00, OR = ∞
Estradiol	*n* = 1 (5% of participants)	*n* = 0 (0% of participants)	*P* = 1.00, OR = ∞
Glucocorticoids	*n* = 3 (15% of participants)	*n* = 0 (0% of participants)	*P* = 0.234, OR = ∞
Lamotrigine	*n* = 0 (0% of participants)	*n* = 1 (6% of participants)	*P* = 0.460, OR = 0
Nonsteroidal anti‐inflammatory drug	*n* = 1 (5% of participants)	*n* = 0 (0% of participants)	*P* = 1.00, OR = ∞
Metamizole	*n* = 0 (0% of participants)	*n* = 1 (6% of participants)	*P* = 0.460, OR = 0
Mirabegron	*n* = 1 (5% of participants)	*n* = 0 (0% of participants)	*P* = 1.00, OR = ∞
Paracetamol	*n* = 1 (5% of participants)	*n* = 0 (0% of participants)	*P* = 1.00, OR = ∞
Proton pump inhibitor	*n* = 4 (20% of participants)	*n* = 3 (18% of participants)	*P* = 1.00, OR = 1.16
Risedronate sodium	*n* = 1 (5% of participants)	*n* = 0 (0% of participants)	*P* = 1.00, OR = ∞
Thyroid hormone replacement	*n* = 1 (5% of participants)	*n* = 1 (6% of participants)	*P* = 1.00, OR = 0.85
Trospiumchlorid	*n* = 1 (5% of participants)	*n* = 1 (6% of participants)	*P* = 1.00, OR = 0.85
5‐alpha reductase inhibitor	*n* = 1 (5% of participants)	*n* = 0 (0% of participants)	*P* = 1.00, OR = ∞
**Changes in medication intake (during intervention)**:			
**Increase in medication dosage**:			
Antidepressants	*n* = 1 (5% of participants)	*n* = 0 (0% of participants)	*P* = 1.00, OR = ∞
Cholinesterase inhibitors	*n* = 2 (10% of participants)	*n* = 0 (0% of participants)	*P* = 0.490, OR = ∞
**Decrease in medication dosage**:			
Glucocorticoids	*n* = 0 (0% of participants)	*n* = 1 (6% of participants)	*P* = 0.460, OR = 0
**New medication(s) and/or medication replacement**:			
Angiotensin‐converting enzyme inhibitors	*n* = 0 (0% of participants)	*n* = 1 (6% of participants)	*P* = 0.460, OR = 0
Antidepressants	*n* = 0 (0% of participants)	*n* = 1 (6% of participants)	*P* = 0.460, OR = 0
Antihistamine	*n* = 1 (5% of participants)	*n* = 0 (0% of participants)	*P* = 1.00, OR = ∞
Phosphodiesterase type 5 (PDE5) inhibitor	*n* = 0 (0% of participants)	*n* = 1 (6% of participants)	*P* = 0.460, OR = 0
**Discontinuation of medication**			
Glucocorticoids	*n* = 1 (5% of participants)	*n* = 0 (0% of participants)	*P* = 1.00, OR = ∞
Statins	*n* = 1 (5% of participants)	*n* = 0 (0% of participants)	*P* = 1.00, OR = ∞
Physiotherapy	n = 2 (10% of participants); median volume^c^ = 45 min/week	*n* = 3 (18% of participants); median volume = 45 min/week	0.644, OR = 0.53
Occupational therapy	*n* = 2 (10% of participants); median volume = 30 min/week	*n* = 2 (12% of participants); median volume = 35 min/week	1.00, OR = 0.84
**Medical training therapy** ^a^	*n* = 3 (15% of participants); median volume^ ^= 90 min/week	*n* = 2 (12% of participants); volume = 110 min/week	1.00, OR = 1.31
**Fitness training** ^b^	*n* = 5 (25% of participants); median volume^ ^= 105 min/week	*n* = 2 (12% of participants); median volume = 175 min/week	0.417, OR = 2.44
(Computerized) cognitive training	*n* = 2 (10% of participants); median volume = 65 min/week	*n* = 4 (24% of participants); volume = 40 min/week	0.383, OR = 0.37
Psychiatric therapy	*n* = 0 (0% of participants);	n = 1 (7% of participants); median volume^c^ = 30 min/week	0.474, OR = ∞

Abbreviations: min, minutes; OR, odds ratio.

^a^ Medical training therapy is prescribed by a doctor and guided and partially supervised by physiotherapists. It typically includes resistance, cardiorespiratory endurance, and balance exercises.

^b^ Fitness training may include the same training content as medical training therapy (i.e., resistance, cardiorespiratory endurance, and balance exercises), but is structured individually and/or with the help of (fitness) coaches.

^c^ Volume = time per training session [minutes] multiplied by frequency of training [times/week].

^d^ *P* values for the between‐group differences tested with Fisher exact test for categorical variables.

* = statistically significant at *P* < 0.05.

#### 3.3.2 Actual delivery of the “Brain‐IT” training

Participants who completed the training performed on average 71.5 ± 26.2 training sessions resulting in an average training volume of 1689 ± 579 minutes over the 12‐week intervention period. On average, 19.5 ± 1.6 training sessions were supervised by our study team. Average attendance and duration adherence rates were 90.0 ± 11.2% and 89.8 ± 12.2%, respectively. The reasons for non‐adherence are summarized in Table 3 and included mainly technical problems and time constraints. Due to technical issues with the exergame device, the feedback mechanism for walking on the spot did not function correctly in a substantial number of participants. As a result, the physical exercise intensity could not be reliably monitored in all participants. Otherwise, no relevant deviations from the “Brain‐IT” training concept were reported.

**TABLE 3:** Summary of reasons for non‐adherence to the “Brain‐IT” training.

		Proportion of occurrence	Proportion of participants affected
Category	Total count	Defined as: proportion of the occurrence of each reason for non‐adherence in relation to the total number of reasons for non‐adherence	Defined as: proportion of participants for whom the following reasons for non‐adherence were reported
**Reasons for non‐attendance adherence**:			
Technical problems	46	38%	*n* = 11; 52% of participants
For time reasons	35	29%	*n* = 13; 62% of participants
Interruptio*n* of training due to adverse events	14	12%	*n* = 2; 10% of participants
Forgot to train	3	2%	*n* = 2; 10% of participants
Comprehension problems	1	1%	*n* = 1; 5% of participants
Other reasons	12	10%	*n* = 4; 19% of participants
Unknown	9	8%	*n* = 4; 19% of participants
**Reasons for non‐duration adherence (i.e., early termination of training sessions)**:			
Accidentally stopped the training (with back plate)	14	40%	*n* = 9; 43% of participants
Technical problems	7	20%	*n* = 4; 19% of participants
Other reasons	4	11%	*n* = 2; 10% of participants
Unknown	10	29%	*n* = 3; 14% of participants

### 3.4 Primary outcome

**FIGURE 2:**
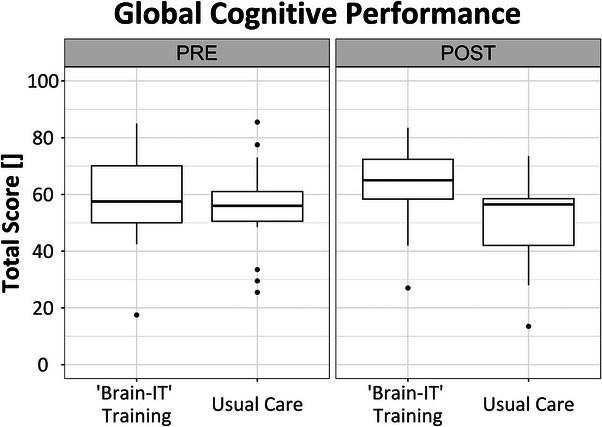
Boxplot for the primary outcome, global cognitive performance, measured with the German version of the Quick Mild Cognitive Impairment screen

Data on the primary outcome is illustrated in Figure 2. The intervention group improved their score in global cognitive performance from 58.7 ± 15.2 points at premeasurements to 62.6 ± 13.5 points at postmeasurements, while the control group showed a decline from 55.1 ± 15.9 points to 51.6 ± 15.3 points. There was a statistically significant effect with a large effect size (F[1, 36] = 8.32, *P* = 0.007, η^2^_p_ [CI_90%_] = 0.197 [0.034, 0.371]) in favor of the intervention group. A post hoc power analysis with G*Power (version 3.1.9.6)^58^ revealed a statistical power of 0.832 for this analysis. Fifty‐five percent of participants in the intervention group and 23% of participants in the control group were responders, showing a clinically relevant improvement in global cognitive performance.

### 3.5 Secondary outcomes

The results of all secondary endpoints are summarized in Table 4 in detail. We observed statistically significant effects with large effect sizes in favor of the intervention group for immediate (F[1, 34] = 5.83, *P* = 0.022, η^2^_p_ [CI_90%_] = 0.154 [0.013, 0.332]) and delayed verbal recall (F[1, 34] = 8.18, *P* = 0.007, η^2^_p_ [CI_90%_] = 0.204 [0.034, 0.382]). The remaining statistical analyses were underpowered and revealed no consistent effects, despite a statistically significant effect with a moderate effect size for a reduction in double support time in favor of the control group (F[1, 34] = 4.96, *p* = 0.033, η^2^_p_ [CI_90%_] = 0.134 [0.006, 0.311]) and a borderline statistically significant effect with a moderate effects size for an improvement of QoL in favor of the intervention group (F[1, 36] = 3.64, *P* = 0.065, η^2^_p_ [CI_90%_] = 0.097 [0, 0.263]).

**TABLE 4:** Statistics for all secondary outcomes.

			Group: “Brain‐IT” training			Group: Usual care					
	Check of assumptions and type of analysis:		PRE‐measurement	POST‐measurement	Sample	PRE‐measurement	POST‐measurement	Sample	ANCOVA statistics:		
Outcome:	All assumptions for parametric analysis met?	Type of analysis	Mean ± SD or median (IQR)	Mean ± SD or median (IQR)	*n*	Mean ± SD or median (IQR)	Mean ± SD or median (IQR)	*n*	*P* value	F Value	η^2^_p_ [90% CI]
**Part 1 ‐ Cognitive Functioning**											
**1.2 Learning and Memory**											
WMS‐IV‐LM Score Part 1 []	✓	parametric	27.7 ± 10.0	29.6 ± 8.7	19^a^	25.1 ± 9.2	23.8 ± 9.9	16^b^	0.022*	5.83	0.154 [0.013, 0.332]
WMS‐IV‐LM Score Part 2 []	x	non‐parametric	8.0 (8.0)	14.0 (14.0)	19^a^	6.5 (9.0)	7.0 (14.5)	16^b^	0.007*	8.18	0.204 [0.034, 0.382]
WMS‐IV‐LM Score Part 2 ‐ Recognition []	✓	parametric	15.8 ± 3.6	16.5 ± 3.2	18^a^, ^c^	16.7 ± 2.6	16.2 ± 2.2	15^b^, ^c^	0.164	2.04	0.064 [0, 0.229]
DSF Total Score []	x	non‐parametric	6.0 (2.0)	6.0 (3.0)	20	7.5 (2.3)	6.5 (2.0)	16^d^	0.865	0.03	0.001 [0, 0.054]
DSF Maximal Span []	x	non‐parametric	5.0 (1.0)	4.0 (1.3)	20	5.0 (2.0)	5.0 (1.3)	16^d^	0.890	0.02	0.001 [0, 0.043]
**1.3 Complex Attention**											
TMT‐A ‐ Completion Time [s]	x	non‐parametric	33.9 (20.7)	35.2 (8.3)	20	31.8 (9.6)	38.9 (17.0)	17	0.270	1.26	0.036 [0, 0.176]
TMT‐A ‐ Number of Errors []	x	non‐parametric	0.0 (2.3)	0.5 (1.3)	20	1.0 (3.0)	0.0 (1.0)	17	0.651	0.21	0.006 [0, 0.104]
TAP Go‐NoGo ‐ RT [ms]	x	non‐parametric	439 (105)	482 (106)	20	412 (140)	448 (112)	17	0.665	0.19	0.006 [0, 0.101]
TAP Go‐NoGo ‐ Number of Errors []	x	non‐parametric	1.5 (3.3)	1.0 (2.0)	20	2.0 (3.0)	1.0 (3.0)	17	0.701	0.15	0.004 [0, 0.095]
**1.4 Executive Functioning**											
HOTAP‐A Combi‐Score [points/min]	x	non‐parametric	4.7 (3.6)	4.6 (3.5)	20	4.7 (3.6)	4.1 (3.5)	16^b^	0.294	1.14	0.033 [0, 0.174]
DSB Total Score []	x	non‐parametric	4.0 (3.0)	3.5 (2.0)	20	4.0 (1.0)	4.5 (1.3)	16^b^	0.434	0.629	0.019 [0, 0.145]
DSB Maximal Span []	x	non‐parametric	3.0 (1.0)	3.0 (1.0)	20	4.0 (1.0)	4.0 (0.3)	16^b^	0.073	3.42	0.094 [0, 0.262]
TMT‐B ‐ Completion Time [s]	x	non‐parametric	97.6 (113.1)	103.0 (78.1)	20	112.4 (81.3)	99.5 (90.0)	17	0.662	0.20	0.006 [0, 0.104]
TMT‐B ‐ Number of Errors []	x	non‐parametric	5.0 (4.0)	6.0 (9.5)	20	5.5 (10.8)	3.5 (9.8)	16^b^	0.381	0.79	0.024 [0, 0.155]
**1.5 Visuospatial Skills**											
MRT ‐ RTs [ms]	x	non‐parametric	4918 (1142)	4761 (1936)	17	3945 (1012)	3778 (2243)	14^c^	0.714	0.14	0.005 [0, 0.109]
MRT ‐ Score []	✓	parametric	42.2 ± 10.6	45.4 ± 10.0	17	44.4 ± 8.6	46.9 ± 8.0	14^c^	0.964	0.00	0.000 [0, 0.000]
**Part 2 ‐ Gait**											
Walking Speed [m ⋅ s^‐1^]	✓	parametric	0.95 ± 0.13	0.95 ± 0.13	18^e^, ^f^	1.02 ± 0.14	0.96 ± 0.18	17	0.248	1.39	0.042 [0, 0.191]
Stride Duration [ms]	x	non‐parametric	1095 (60)	1065 (85)	18^e^, ^f^	1060 (140)	1060 (130)	17	0.620	0.25	0.008 [0, 0.115]
Stride Length [cm]	✓	parametric	102.8 ± 11.2	102.2 ± 11.2	18^e^, ^f^	107.8 ± 14.5	102.5 ± 14.9	17	0.178	1.89	0.056 [0, 0.213]
Stance Phase Duration [ms]	✓	parametric	613.1 ± 67.4	611.8 ± 69.9	18^e^, ^f^	645.5 ± 94.7	617.0 ± 117.0	17	0.258	1.33	0.040 [0, 0.188]
Swing Phase Duration [ms]	✓	parametric	415.3 ± 50.6	409.9 ± 46.7	18^e^, ^f^	432.8 ± 56.0	418.8 ± 63.8	17	0.644	0.22	0.007 [0, 0.110]
Single Support Time [ms]	✓	parametric	414.7 ± 51.4	409.8 ± 47.7	18^e^, ^f^	433.5 ± 56.6	419.9 ± 63.3	17	0.669	0.19	0.006 [0, 0.106]
Double Support Time [ms]	x	non‐parametric	98.7 (27.4)	98.1 (21.9)	18^e^, ^f^	101.2 (19.1)	90.4 (23.4)	17	0.033*	4.96	0.134 [0, 0.311]
**Part 3 ‐ IADL**											
T‐Score []	✓	parametric	56.5 (8.5)	58.1 (8.9)	18^g^	52.8 (7.9)	55.4 (7.2)	17	0.990	0.00	0.000 [0, 0.000]
**Part 4 ‐ Psychosocial Factors**											
Quality of Life (QoL‐AD) []	x	non‐parametric	39.0 (5.5)	39.0 (4.3)	20	38.0 (6.0)	38.0 (7.0)	17	0.065	3.64	0.097 [0, 0.263]
DASS‐21 ‐ Depression []	x	non‐parametric	2.0 (5.0)	1.5 (4.0)	20	2.0 (4.0)	1.0 (4.0)	17	0.993	0.00	0.000 [0, 0.000]
DASS‐21 ‐ Anxiety []	x	non‐parametric	2.5 (3.3)	1.5 (3.3)	20	1.0 (2.0)	1.0 (2.0)	17	0.996	0.00	0.000 [0, 0.000]
DASS‐21 ‐ Stress []	x	non‐parametric	3.0 (5.0)	4.0 (3.5)	20	4.0 (6.0)	4.0 (4.0)	17	0.279	1.212	0.000 [0, 0.174]
**Part 5 ‐ Heart‐Rate Variability**											
mRR [ms]	x	non‐parametric	873 (121)	851 (154)	14^f^, ^h^	773 (186)	776 (190)	12^f^, ^h^	0.741	0.112	0.005 [0, 0.270]
RMSSD [ms]	x	non‐parametric	16.7 (25.7)	13.2 (20.1)	14^f^, ^h^	20.3 (13.7)	21.1 (25.9)	12^f^, ^h^	0.591	0.297	0.013 [0, 0.157]
pNN50 [%]	x	non‐parametric	1.5 (16.1)	0.4 (7.1)	14^f^, ^h^	1.4 (5.6)	1.3 (17.2)	12^f^, ^h^	0.531	0.404	0.017 [0, 0.170]
HF [ms^2^]	x	non‐parametric	89.0 (452.9)	79.5 (109.8)	14^f^, ^h^	155.5 (156.0)	155.5 (371.5)	12^f^, ^h^	0.230	1.524	0.062 [0, 0.251]
HFnu [nu]	x	non‐parametric	40.5 (28.0)	36.3 (32.9)	14^f^, ^h^	54.2 (33.9)	65.8 (25.4)	12^f^, ^h^	0.126	2.525	0.099 [0, 0.298]
SD1 [ms]	x	non‐parametric	11.8 (18.1)	9.4 (14.2)	14^f^, ^h^	14.4 (9.8)	14.9 (18.3)	12^f^, ^h^	0.600	0.283	0.012 [0, 0.155]
PNS‐Index []	x	non‐parametric	–0.67 (1.72)	–1.05 (1.01)	14^f^, ^h^	–1.06 (1.47)	–1.03 (1.99)	12^f^, ^h^	0.772	0.086	0.004 [0, 0.111]

*Notes*: Normality distribution of data was checked using the Shapiro–Wilk test and Q‐Q‐plots. The level of significance was set to *P* ≤ 0.05 (two‐sided, uncorrected). The assumption of homogeneity of variance was checked using a Levene test. In case all assumptions for analysis of covariance (ANCOVA) were met, effects of the addition of the “Brain‐IT” training concept to usual care compared to usual care were analyzed using an ANCOVA with the premeasurement value as a covariate for the predicting group factor and the postmeasurement value as an outcome variable [1]. In case not all assumptions were met, a Quade non‐parametric ANCOVA was used. To discover whether effects are substantive, partial eta‐squared (η^2^_p_) effect sizes were calculated for all primary and secondary outcomes. Effect sizes were interpreted to be small (0.01 ≤ η^2^_p_ < 0.06), medium (0.06 ≤ η^2^_p_ < 0.14), or large (η^2^_p_ > 0.14) [2].

Abbreviations: ANCOVA, analysis of covariance; DASS‐21, Depression, Anxiety and Stress Scale‐21; DSB, Digit Span Backward; DSF, Digit Span Forward; HOTAP‐A, HOTAP picture‐sorting test part A; IADL, Instrumental Activities of Daily Living; IQR, interquartile range; MRI, magnetic resonance imaging; MRT, Mental Rotation Task; n, sample size; PEBL, Psychology Experiment Building laLguage; QMCI, Quick Mild Cognitive Impairment Screen; QoL‐AD, Quality of Life‐Alzheimer's Disease; SD, standard deviation; TAP Alertness, subtest “Alertness” of the Test of Attentional Performance; TAP Go‐NoGo, subtest “Go‐NoGo” of the Test of Attentional Performance; TAP Incompatibility, subtest “Incompatibility” of the Test of Attentional Performance; TMT‐A and B, Trail Making Test Part A and B; vm‐HRV, vagally‐mediated heart‐rate variability; WMS‐IV‐LM, subtest ‘logical memory’ of the Wechsler Memory Scale‐ fourth edition; η^2^_p_ [90% CI], partial eta‐squared [90% confidence interval].

^a^ Missing data because measurement was aborted due to emotional breakdown.

^b^ Missing data because measurement was not performed due to the psychological/emotional overload of the participant.

^c^ Missing data because measurement was not performed due to lack of test comprehension.

^d^ Missing data because measurement was aborted because the maximum processing time according to the test instructions was reached.

^e^ Missing data because measurement was not performed because participant had knee surgery a few weeks ago and is still undergoing physical therapy, which could introduce bias into the analysis of gait changes over time.

^f^ Missing data due to technical problems with the measurement device.

^g^ Missing data due to unavailability of a close relative of the study participant.

^h^ Missing data due to insufficient data quality.

References: 1. Field A, Miles J, Field Z. Discovering statistics using R. Sage publications; 2012.

2. Cohen J. Statistical power analysis for the behavioral sciences; ISBN: 1134742703. Routledge; 1988.

* = statistically significant at P < 0.05.

## 4 DISCUSSION

This RCT investigated the effectiveness of combining exergame‐based motor‐cognitive training with HRV‐BF delivered through an individualized training concept called “Brain‐IT.” The results provide robust evidence that “Brain‐IT” training is effective for enhancing global cognitive performance, immediate verbal recall, and delayed verbal recall. However, the results regarding near‐ and far‐transfer effects were inconclusive.

### 4.1 Principal findings

This was the first RCT to examine the effectiveness of combining exergame‐based motor‐cognitive training with HRV‐BF. Therefore, comparisons with previous literature are limited. To the best of our knowledge, there are no studies available that investigated the effects of HRV‐BF on cognitive performance in individuals with mNCD so far. Pooled evidence from studies investigating the effects of exergaming in individuals with mNCD found small^11^ to medium^59^ effects favoring the intervention, which is consistent with pooled evidence of simultaneous motor‐cognitive training, which also reported small^11^, ^60^ to medium^61^, ^62^ effect sizes. Based on the large effect size observed, it appears that our exergame‐based training may be more effective than previously investigated training concepts, which aligns with the findings of Swinnen et al. in institutionalized patients with major neurocognitive disorders.^63^ In addition, we found that 23% of the participants in the control group were responders, which is consistent with the spontaneous rate of reversion that can be expected in this population.^64^ However, a substantially larger proportion of participants showed a clinically relevant improvement in response to the training.

These observations may be reflective of our rigorous methodology and the quality of its resulting training concept. Only one previous study applied exergame training that individually prescribed content on the basis of a patient's cognitive abilities in mNCD.^65^ In contrast, we investigated an evidence‐based, purpose‐designed and user‐centered training concept that was iteratively co‐designed, tested, and refined with continuous patient and public involvement that is individualized and progressed according to predefined progression rules on different levels and that includes a number of elements and support strategies to facilitate training motivation and self‐determined training behavior. Therefore, it seems reasonable to conclude that this extensive groundwork has paid off by showing larger‐than‐expected effects on the outcomes for which the training was primarily designed. However, it is possible that these effects are partially attributable to our novel intervention approach with the addition of HRV‐BF, which may have induced positive synergistic effects.

The literature explains that HRV‐BF training increases cardiac autonomic control, which in turn increases vagal afferent transmission to the forebrain and activates brain networks such as the central autonomous network, including the prefrontal cortex. This activation is important for self‐regulation and the control of cognitive processes, and helps to restore hemostasis.^18^, ^19^, ^20^ More specifically, electroencephalography studies indicate an increase in alpha power and a decrease in theta power, as well as increased levels of oxygenated hemoglobin in the anterior part of the prefrontal cortex as measured by near‐infrared spectroscopy. Furthermore, a study using functional MRI has shown increased activity in cortical areas such as the prefrontal, motor, and parietal cortices, as well as subcortical structures including the pons, thalamus, subparabrachial nucleus, periaqueductal gray, and hypothalamus.^20^ The observed effects of HRV‐BF on improving cognitive performance, particularly executive function, have been attributed to these neurophysiological changes.^20^, ^22^, ^23^

Based on the increased activation and oxygenation of brain regions relevant for cognitive adaptations, it could be hypothesized that HRV‐BF enhances receptivity for neuroplastic changes induced by physical and/or cognitive training when combined with simultaneous motor‐cognitive training, similar to the facilitation effects on cognitive adaptations in response to cognitive training performed simultaneously with at least moderate intensity physical exercise.^12^, ^13^, ^14^, ^66^ However, this hypothesis needs to be specifically tested in future research.

This lack of consistent transfer effects observed in this study may be due to the higher‐than‐expected baseline performance. Specifically, our study participants' performance was closer to the reference values of healthy older adults than to mNCD for several outcomes, including complex attention (i.e., TMT‐A,^67^ TAP Go‐NoGo^68^), executive function (i.e., TMT‐B^67^), and spatiotemporal parameter of gait.^69^ We also observed bottom effects for symptoms of depression, anxiety, and stress, which are comparable to the reference values.^70^ Consequently, room for improvements in these endpoints was limited. These observations could be explained by a potential selection bias in the recruitment process, as the main reason for non‐participation was patient declination (65% of reasons for non‐participation). Individuals with prominent executive deficits and/or gait insecurities may lack confidence in their ability to commit to the study and the 12 weeks of regular and partially independent training it entails. Therefore, future research should aim to implement strategies that further reduce participation barriers for these individuals.

### 4.2 Implications for research and clinical practice

Larger confirmatory RCTs are necessary to draw conclusions about the potential near‐ and far‐transfer effects of the training. Additionally, it is recommended to compare the training with active comparators, including isolated and combined physical and/or cognitive exercises,^53^ as well as HRV‐BF training. Furthermore, future research should investigate whether the observed improvements in cognitive performance translate to affecting the rates of progression to or onset of dementia, which is essentially what should be achieved by effective secondary prevention.^8^

In the next step, the implementation of the “Brain‐IT” training concept in clinical practice should be tested. In this regard, it is recommended to adapt and implement the training concept with various hardware and software solutions to overcome current barriers and to further develop and improve technologies to provide an optimal training environment and stimuli^38^ in line with a “training first” approach.^53^ Consistent with this approach, our training concept was reported using CERT^45^ and provides detailed guidance on how to adapt it to other hardware and software solutions. Therefore, the “Brain‐IT” training concept can and should be incorporated as an adjunctive therapy to standard care for improving global cognitive performance and memory of individuals with mNCD.

Previous studies have reported potential neurophysiological benefits induced by exergaming, but further research is required in this area.^71^, ^72^, ^73^ Therefore, it is necessary to further elucidate the underlying biological mechanisms of action. These investigations should then guide further research aimed at further improving the training by providing optimal stimuli to influence the pathological mechanisms of mNCD and ultimately maximize the training's effectiveness in the secondary prevention of mNCD.

### 4.3 Strengths and limitations

This RCT had several important strengths. First, the evaluated intervention targets various mechanisms of action to alleviate the pathological state in individuals with mNCD and has been iteratively designed, developed, and evaluated following best practice recommendations of the Medical Research Council^35^ as well as the MIDE framework^36^ with continuous patient and public involvement^34^, ^37^ (see Manser et al.^34^ for the project's methodology). Second, the study as well as its intervention were strictly planned, conducted, and reported in accordance with established guidelines including the SPIRIT,^41^, ^42^ CONSORT,^43^ as well as CERT,^45^ to ensure full reproducibility. The study also adhered to several best practice characteristics for exercise/exergame studies, such as blinding of outcome assessors, allocation concealment, modified intention‐to‐treat analysis, and reporting of all relevant study characteristics to minimize the risk of bias.^53^, ^74^ Third, the study was preregistered at ClinicalTrials.gov (NCT05387057), we submitted the study protocol for publication before starting recruitment,^39^ and transparently reported any deviations from the published study protocol. Finally, we included various etiologies of mNCD and chose relatively broad eligibility criteria to increase the generalizability of our findings.

The study also has some key limitations that are worth mentioning. First, we originally aimed to only explore the effectiveness of the addition of the “Brain‐IT” training concept to usual care. In line with this, the sample size for this RCT was justified to provide a sufficiently precise estimate of the treatment effect to minimize the sample size needed for a future full‐scale RCT.^54^ However, we obtained effect sizes for the primary outcome, as well as for immediate and delayed verbal recall, that were larger than expected. This prompted us to conduct a post hoc power analysis, which confirmed that the statistical analysis for these outcomes was sufficiently powered. Second, our investigation of the addition of the “Brain‐IT” training to usual care was limited by the fact that the (memory) clinics where participants were recruited provided the usual care interventions, making it impossible to standardize contact times. This may have impacted some of our findings. However, comparing an intervention against treatment as usual is a recommended practice for effectiveness studies.^35^ Third, usual care interventions were assessed by self‐report of patients. To mitigate possible biased information, the study team posed specific questions regarding participants' engagement in typical care interventions and actively involved their proxies in collecting this information. Fourth, patients screened for MCI according to predefined criteria were recruited in addition to patients with a clinical diagnosis of mNCD, which increased the heterogeneity of the study population. However, this is in line with the project's objective to investigate an intervention not only to treat clinically diagnosed patients with mNCD but also to prevent progression to dementia in individuals at risk who might not have been diagnosed (yet). Fifth, women were underrepresented, which might limit the generalizability of our findings. Finally, medication changes during the intervention could have potentially introduced bias. Although we did not find any statistically significant differences between the groups in terms of pharmacological or non‐pharmacological usual care interventions, 10% of participants in the intervention group had an increase in their dose of cholinesterase inhibitors and the control group had a substantially larger proportion of participants taking cholinesterase inhibitors throughout the study. Because the evidence for cholinesterase inhibitors suggests only a stabilization or slowing of cognitive decline, not an improvement in cognition as observed in the intervention group,^75^ this confounding effect played a subordinate role.

## 5 CONCLUSION

The combination of exergame‐based motor‐cognitive training with HRV‐BF delivered via an individualized exergame‐based training concept (called “Brain‐IT”) is effective in improving global cognitive performance, immediate verbal recall, and delayed verbal recall. Confirmatory RCTs with larger sample sizes are necessary to draw conclusions about the potential near‐ and far‐transfer effects of the training and to investigate whether the observed improvements in cognitive performance translate to affecting the rates of progression to or onset of dementia. Additionally, the training should be compared to different active comparators, such as isolated and combined physical and cognitive exercises or HRV‐BF training. Future research should also test the implementation of the training in clinical practice and further optimize the “Brain‐IT” training concept. In this regard, the underlying biological mechanisms of action should be elucidated to guide further research aimed at further improving the training by providing optimal stimuli to influence the pathological mechanisms of mNCD and ultimately maximize its effectiveness in the secondary prevention of mNCD.

## AUTHOR CONTRIBUTIONS

Patrick Manser was responsible for the conception of the study under the supervision of Eling D. de Bruin. Patrick Manser was responsible for participant recruitment, data collection, statistical analysis, and writing the manuscript. Both authors contributed to the revisions of the manuscript, and read and approved the submitted version of the manuscript.

## CONFLICT OF INTERESTS STATEMENT

The authors declare no conflicts of interest. Author disclosures are available in the supporting information.

## CONSENT STATEMENT

All the study procedures were performed in accordance with the Declaration of Helsinki. The study protocol was approved by the ethics committees of Zurich and Eastern Switzerland (EK‐2022‐00386).

## CLINICAL TRIAL REGISTRATION

Trial Registration: ClinicalTrials.gov NCT05387057; https://clinicaltrials.gov/ct2/show/NCT05387057

## Supporting information

Supporting Information

Supporting Information

## Data Availability

The data sets that were generated and analyzed during the current study are available in the Zenodo repository at https://doi.org/10.5281/zenodo.10695988.
