# Supplementary material for: “Brain‐IT”: Exergame training with biofeedback breathing in neurocognitive disorders
Source: Alzheimers Dement. 2024 May 29;20(7):4747–64. doi: 10.1002/alz.13913 (PMC11247687; doi:10.1002/alz.13913)
Supplement: Supplementary file 1 — Supporting Information [file ALZ-20-4747-s002.pdf]

Supplementary Files to Publication:

'Brain-IT' - exergame training with biofeedback breathing in neurocognitive disorders

Patrick Manser[a]* and Eling D. de Bruin[a-c]

[a] ETH Zurich, Department of Health Sciences and Technology, Institute of Human Movement Sciences and Sport, Motor Control and Learning Group, Leopold-Ruzicka-Weg 4, 8093 Zurich, Switzerland

[b] OST - Eastern Swiss University of Applied Sciences, Department of Health, Rosenbergstrasse 59, 9001 St.Gallen, Switzerland;

[c] Karolinska Institute, Department of Neurobiology, Care Sciences and Society, Division of Physiotherapy, Alfred Nobels Alle 23, 141 83 Huddinge, Sweden

16-digit ORCID and e-mail of the author(s):

Patrick Manser	ORCID: 0000-0003-3300-6524	patrick.manser@hest.ethz.ch
Eling D. de Bruin	ORCID: 0000-0002-6542-7385	eling.debruin@hest.ethz.ch

***Correspondence:**

Patrick Manser
ETH Zurich | Department of Health Sciences and Technology
Institute of Human Movement Sciences and Sport
Motor Control and Learning Group
HCP H24.3
Leopold-Ruzicka-Weg 4 | 8093 Zurich | Switzerland
Tel.: +41 79 519 96 46
E-mail: patrick.manser@hest.ethz.ch

1 Supplementary File 1 - CONSORT checklist

Table 1: 2017 CONSORT Checklist of Information to Include When Reporting Randomized Trials Assessing nonpharmacologic treatments [1]

Section/Topic:	Item No:	Checklist item:	Reported in section(s):
**TITLE AND ABSTRACT:**			
	1a	Identification as a randomized trial in the title.	Journal restrictions on the number of characters in the title did not allow this information to be included in the title. Therefore, it is reported in the 'Abstract' section.
	1b	Structured summary of trial design, methods, results, and conclusions (for specific guidance see CONSORT for abstracts).	‘Abstract’
**INTRODUCTION:**			
Background and objectives:	2a	Scientific background and explanation of rationale.	‘Introduction’ and ‘Materials and Methods - Prior Work’
	2b	Specific objectives or hypotheses.	‘Materials and Methods - Objectives and Hypotheses’; more details in the published study protocol [42]
**METHODS:**			
Trial design:	3a	Description of trial design (such as parallel, factorial) including allocation ratio. When applicable, how care providers were allocated to each trial group.	‘Materials and Methods - Overview of the Trial Design, Participants, and Interventions’; more details in the published study protocol [42]
	3b	Important changes to methods after trial commencement (such as eligibility criteria), with reasons.	‘Materials and Methods - Protocol and Registration - Important changes to the trial design and study setting after commencement’; more details in the published study protocol [42]
Participants:	4a	Eligibility criteria for participants. When applicable, eligibility criteria for centers and for care providers.	‘Materials and Methods - Overview of the Trial Design, Participants, and Interventions’; more details in the published study protocol [42]
	4b	Settings and locations where the data were collected.	‘Materials and Methods - Overview of the Trial Design, Participants, and Interventions’; more details in the published study protocol [42]
Interventions:	5	The interventions for each group with sufficient details to allow replication, including how and when they were actually administered. Precise details of both the experimental treatment and comparator.	‘Materials and Methods - Overview of the Trial Design, Participants, and Interventions’; more details in the published study protocol [42] and supplementary file 2
	5a	Description of the different components of the interventions and, when applicable, description of the procedure for tailoring the interventions to individual participants.	‘Materials and Methods - Overview of the Trial Design, Participants, and Interventions’; more details in the published study protocol [42] and supplementary file 2
	5b	Details of whether and how the interventions were standardized.	‘Materials and Methods - Overview of the Trial Design, Participants, and Interventions’; more details in the published study protocol [42] and supplementary file 2
	5c	Details of whether and how adherence of care providers to the protocol was assessed or enhanced.	N/A

	5d	Details of whether and how adherence of participants to interventions was assessed or enhanced.	'Materials and Methods - Overview of Outcomes - Other Endpoints - Adherence Protocol'
Outcomes:	6a	Completely defined pre-specified primary and secondary outcome measures, including how and when they were assessed.	'Materials and Methods - Overview of Outcomes'; more details in the published study protocol [42]
	6b	Any changes to trial outcomes after the trial commenced, with reasons.	N/A
Sample size:	7a	How sample size was determined. When applicable, details of whether and how the clustering by care providers or centers was addressed.	'Materials and Methods - Sample Size'; more details in the published study protocol [42]
	7b	When applicable, explanation of any interim analyses and stopping guidelines.	N/A (see 'Materials and Methods - Statistical Methods'
Randomization:			
Sequence generation:	8a	Method used to generate the random allocation sequence.	'Materials and Methods - Randomization'; more details in the published study protocol [42]
	8b	Type of randomization; details of any restriction (such as blocking and block size).	'Materials and Methods - Randomization'; more details in the published study protocol [42]
Allocation concealment mechanism:	9	Mechanism used to implement the random allocation sequence (such as sequentially numbered containers), describing any steps taken to conceal the sequence until interventions were assigned.	'Materials and Methods - Randomization'; more details in the published study protocol [42]
Implementation:	10	Who generated the random allocation sequence, who enrolled participants, and who assigned participants to Interventions.	'Materials and Methods - Randomization'; more details in the published study protocol [42]
Blinding:	11a	If done, who was blinded after assignment to interventions (e.g., participants, care providers, those administering co-interventions, those assessing outcomes) and how.	'Materials and Methods - Blinding'; more details in the published study protocol [42]
	11b	If relevant, description of the similarity of interventions.	N/A
	11c	If blinding was not possible, description of any attempts to limit bias	N/A
Statistical methods:	12a	Statistical methods used to compare groups for primary and secondary outcomes. When applicable, details of whether and how the clustering by care providers or centers was addressed.	'Materials and Methods - Statistical Methods'
	12b	Methods for additional analyses, such as subgroup analyses and adjusted analyses	N/A (see 'Materials and Methods - Statistical Methods'
**RESULTS:**			
Participant flow (a diagram is strongly recommended):	13a	For each group, the numbers of participants who were randomly assigned, received intended treatment, and were analyzed for the primary outcome. The number of care providers or centers performing the intervention in each group and the number of patients treated by each care provider or in each center.	'Results - Recruitment and Participant Flow'
	13b	For each group, losses and exclusions after randomization, together with reasons.	'Results - Recruitment and Participant Flow'
	13c	For each group, the delay between randomization and the initiation of the intervention.	'Results - Recruitment and Participant Flow'
	13d	Details of the experimental treatment and comparator as they were implemented.	'Results - Delivery of the interventions'
Recruitment:	14a	Dates defining the periods of recruitment and follow-up.	'Results - Recruitment and Participant Flow'

	14b	Why the trial ended or was stopped.	‘Results - Recruitment and Participant Flow’
Baseline data:	15	A table showing baseline demographic and clinical characteristics for each group. When applicable, a description of care providers (case volume, qualification, expertise, etc.) and centers (volume) in each group.	‘Results - Baseline Data’
Numbers analyzed:	16	For each group, number of participants (denominator) included in each analysis and whether the analysis was by original assigned groups.	Primary Outcome: ‘Results - Recruitment and Participant Flow’ and ‘Results – Primary Outcome’<br><br>Secondary Outcomes: ‘Results – Secondary Outcomes’ and ‘Table 3: Statistics for all secondary outcomes’
Outcomes and estimation:	17a	For each primary and secondary outcome, results for each group, and the estimated effect size and its<br>precision (such as 95% confidence interval).	Primary Outcome: ‘Results – Primary Outcome’<br><br>Secondary Outcomes: ‘Results – Secondary Outcomes’ and ‘Table 3: Statistics for all secondary outcomes’
	17b	For binary outcomes, presentation of both absolute and relative effect sizes is recommended	N/A
Ancillary analyses:	18	Results of any other analyses performed, including subgroup analyses and adjusted analyses, distinguishing pre-specified from exploratory	N/A (see ‘Materials and Methods - Statistical Methods’
Harms:	19	All important harms or unintended effects in each group (for specific guidance see CONSORT for harms)	‘Results - Recruitment and Participant Flow’
**DISCUSSION:**			
Limitations:	20	Trial limitations, addressing sources of potential bias, imprecision, and, if relevant, multiplicity of analyses. In addition, take into account the choice of the comparator, lack of or partial blinding, and unequal expertise of care providers or centers in each group.	‘Discussion - Strength and Limitations’
Generalizability:	21	Generalizability (external validity) of the trial findings according to the intervention, comparators, patients, and care providers and centers involved in the trial.	‘Discussion - Principal Findings’ and ‘Discussion - Strength and Limitations’
Interpretation:	22	Interpretation consistent with results, balancing benefits - and harms, and considering other relevant evidence.	‘Discussion - Principal Findings’ and ‘Discussion - Implications for Research and Clinical Practice’
**OTHER INFORMATION:**			
Registration:	23	Registration number and name of trial registry.	‘Abstract’ and ‘Materials and Methods - Overview of the Trial Design, Participants, and Interventions’
Protocol:	24	Where the full trial protocol can be accessed, if available.	‘Materials and Methods - Protocol and Registration’
Funding:	25	Sources of funding and other support (such as supply of drugs), role of funders.	‘Sources of Funding’

2 Supplementary File 2 - refined ‘Brain-IT’ training concept

2.1 Introduction

This training concept has been developed in the project ‘Brain-IT’. In this project, we designed and developed a novel training concept (‘Brain-IT’) specifically for older adults with mild neurocognitive disorder (mNCD). The ‘Brain-IT’ training concept represents a guideline for applying a combination of exergame-based motor-cognitive training and resonance breathing guided by heart rate variability biofeedback (HRV-BF) training by standardizing the training characteristics (e.g., training frequency, intensity, and duration), as well as the structure and content of training, whereas the exergame device and the specific games used within each of the defined neurocognitive domains can be replaced by alternative exergames.

The projects’ methodology [2] followed the guidelines of the Medical Research Council for the development and evaluation of complex interventions [3] as well as the Multidisciplinary Iterative Design of Exergames (MIDE) – Framework [4]. The ‘Brain-IT’ project was structured in three phases. In phase 1, we systematically combined a comprehensive literature synthesis [5] with qualitative research including primary end users (older adults with mNCD), secondary end users (physiotherapists, occupational therapists, healthcare professionals), exergaming researchers, as well as experts from the exergaming industry [6] to specify a set of design requirements for the ‘Brain-IT’ training concept. In phase 2, possible concepts for the exergame-based training concept were co-designed and elaborated based on the set of design requirements defined in phase 1. The first prototype of the resulting ‘Brain-IT’ training concept [5] then entered the iterative cycle of feasibility, usability, safety and acceptance testing and integrating study results for further development based on co-design until an "acceptable" solution was achieved. The results of this process revealed that the resulting ‘Brain-IT’ training is feasible, usable, safe, and highly accepted by older adults with mNCD and preliminary data on the effects of the ‘Brain-IT’ training are promising [7]. The results on the effectiveness of the ‘Brain-IT' concept were published with the accompanied paper (study protocol [8]; registered at clinicaltrials.gov prior to the start of patients’ recruitment (NCT05387057)).

In the ‘Brain-IT’ project, we used technology of Dividat AG (i.e., ‘Senso Flex’ (Dividat AG, Schindellegi, Switzerland; hardware: prototype version 2, software: version 22.4.0-360-

gf9df00d5b), Polar (i.e., heart rate monitor (Polar M430) and sensor (Polar H10)), and Kubios (Kubios HRV Premium (Kubios Oy, Kuopio, Finland, version 3.4)) to implement our training concept. To ensure replicability, the ‘Brain-IT’ training concept was planned and reported according to the Consensus on Exercise Reporting Template (CERT) [9] and provides specific instructions on how to adapt the ‘Brain-IT’ training concept to other hardware and software solutions.

2.2 Overview of the Exercise and Training Variables

**F**requency:	≥ 5x/week
**I**ntensity/Complexity:	monitored and adapted according to predefined progression rules (section 2.4).
**T**ype & Specificity:	combination of exergame-based simultaneous motor-cognitive training with incorporated cognitive tasks and HRV-BF training that is adopted with an individualized (deficit-oriented) focus on (1) learning and memory, (2) executive function, (3) complex attention, and (4) visuo-spatial skills.
**T**ime & Duration:	≥ 24 min/session for ≥ 12 weeks
**V**olume:	≥ 120 min/week
**V**ariability:	according to the concept of MYCHOICE (section 2.8).
**P**rogression:	according to predefined progression rules (section 2.7).
**D**ensity:	In general, training sessions should be performed on different days. If multiple sessions are performed on the same day, recovery time between sessions should be ≥ 4 hours.
**L**ocation:	at participant's homes
**G**uidance & Supervision:	structured in 3 phases starting with a guided familiarization period with the aim to lead participants to being able to train independently in the long-term.

2.3 Overview of the Training

2.3.1 Implementation in the Project ‘Brain-IT’:

The ‘Brain-IT’ training concept consists of an individualized combination of exergame-based simultaneous motor-cognitive training with incorporated cognitive tasks and HRV-BF training that is adopted with an individualized (deficit-oriented) focus on (1) learning and memory, (2) executive function, (3) complex attention, and (4) visuo-spatial skills. According to the training concept, each participant is instructed to train ≥ 5x/week for ≥ 24 min per session resulting in a weekly training volume of ≥ 120 min. All training sessions are planned to take place at participant’s homes using the exergame training system Senso Flex. In case a participant prefers to train at one of the study centers or has not enough space for training with the ‘Senso Flex’ at home, the participants can be instructed to train with an adapted training frequency of ≥ 3x/week for 24 min per session at one of the study sites using the exergame training system ‘Senso’ (Dividat AG, Schindellegi, Switzerland; CE certification), but it is still recommended to train at the suggested optimal frequency (≥ 5x/week) and volume (≥ 120 min/week).

The ‘Senso Flex’ is a home-based version of the ‘Senso’. The ‘Senso’ was developed for stationary use in physiotherapies, nursing homes, or rehabilitation clinics. It consists of a 1.13 m × 1.13 m robust stepping platform built from metal and glass including a handrail for balance support. The stepping platform is connected to a computer and a frontal television screen. In contrast, ‘Senso Flex’ was developed for home-based use and consists of a 1.11 m x 0.99 m rollable mat as stepping platform that is plugged into the portable computer and a television (or other screen) at home) and can be packed up and put away after training. In both cases, the pressure-sensitive stepping platform is divided into five areas: (1) center (home position), (2) front, (3) right, (4) back, and (5) left. The device detects participants’ position and timing of movements (including weight shifting, walking on the spot, and steps in four directions: front, right, back, and left) to interact with different game scenarios, that are programmed in the Dividat training software (i.e. the same training software is used for both types of stepping platforms (‘Senso’ and ‘Senso Flex’)). Weight-shifting, walking on the spot, and stepping movements to the four directions enable the interaction and control of the virtual exergame scenarios that are displayed on a screen right in front of the participant. Visual, auditory and

somatosensory (vibrating platform; only available on the ‘Senso’) feedback is provided in real-time in order to enrich the game experience.

The training intervention starts with a familiarization period of two weeks. During this phase, most of the training sessions (i.e. 4 out of 5 sessions) are supervised by our research team. After this initial guided familiarization period, supervision of training sessions is gradually reduced to 1x/week during a four-week transition phase. This transition phase aims to lead participants to being able to train independently. In this transition phase, the amount of supervision of training sessions is individually determined within a predefined range (see Figure 1) in accordance with the capabilities and preferences of the participants. From the 7th week until completion of the training intervention, semi-autonomous training with one supervised training session per week is prescribed for each participant. During independent training sessions, the research team is available by phone to provide help when needed. In case the training sessions need to take place at one of the study sites using the exergame training system ‘Senso’, the absolute amount of supervision is kept the same, since participants are instructed to train at ≥ 3x/week but it is still recommended to train at the suggested optimal frequency (≥ 5x/week) and volume (≥ 120 min/week).

2.3.2 How to adapt the ‘Brain-IT’ training concept to other hardware and software solutions:

Step 1: Select an exergame device that meets the following criteria:

Step 1a: If you intend to develop a new exergame system to implement the ‘Brain-IT’ training, follow all the steps of the MIDE-framework [4]. A detailed description of the methodology and the integration of the MIDE-framework into the ‘Brain-IT’ project can be found in [5].

Step 1b: If you intend to use an existing exergame system to implement the ‘Brain-IT’ training, make sure it meets all of the following criteria:

- General Criteria:
    - Step-based exergame played in standing position (including treadmill-based exergames)
    - Device is (optimally) suitable for home use.

    - Exergame device provides real-time visual, auditory and/or tactile feedback (i.e. multisensory feedback to be used as a positive reinforcement mechanism)

- Safety requirements:
    - The exergame system provides a handrail or similar for balance support or can be combined with an external balance support device (e.g., walking sticks, harness, mobile handrails) to prevent falls (especially at the beginning of training).

- Usability requirements:
    - Ensure good usability of the exergames. As a rule of thumb, each participant should be able to use the system independently (including setting up and starting training) after the two-week familiarization phase and with the help of a user manual.

- Basic requirements to game design:
    - Provide simple graphics and ensure good contrast (i.e., main task located in the center of the screen AND only elements that are related to the game task are visible).
    - Provide game tasks with a certain closeness to everyday life.
    - Provide easily comprehensible and clearly designed tasks.
    - Avoid unexpected appearings or technical problems.
    - Avoid confronting performance feedback by providing very subtle negative feedback in case of mistakes to help ensure task comprehension.

2.4 Structure of each Exergame Session

2.4.1 Implementation in the Project 'Brain-IT':

Throughout the training intervention period, all sessions are prescribed following the same basic structure (see Figure 1). Each session consists of three blocks with 3 phases per block.

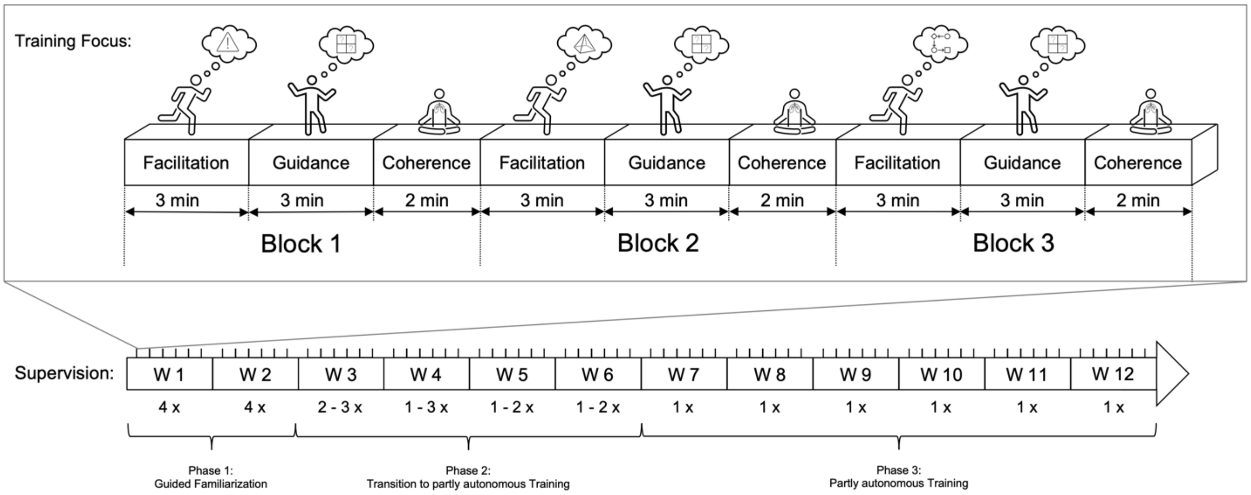

Figure 1: Overview of the exergame-based intervention concept and the basic structure of each exergame session (here as an example for a patient with amnestic-single domain mild neurocognitive disorder with a training focus on learning and memory in week 1).

<u>Phase 1 - Facilitation</u>

Phase 1 - Facilitation aims to apply a moderate physical intensity in the context of challenging but feasible neurocognitive and motoric demands mainly intending to *"trigger neurophysiological mechanisms, which promote neuroplasticity"* [10, 11] while additionally using *"cognitive stimulation […] to "guide" these neuroplastic processes"* [10-12]. This phase includes games focusing on neurocognitive domains that are least impaired. The external task demands is individually adapted to ensure an appropriate internal training load. More specifically, the internal training load is subdivided into a fixed component (i.e. physical intensity) and a variable component (i.e. neurocognitive (game-) demand). An additional stepping task is used to set the level of physical intensity. It includes walking on the spot at a predefined stepping frequency that is needed to reach a moderate level of physical intensity (i.e. ranging between 40 and 59 % heart rate reserve (HRR) [13])). The stepping frequency is individually determined for each participant (see section 2.7). A battery figure add-on is visible in the center of the screen that provides real-time visual feedback whether the

predefined stepping frequency is reached. More specifically, if the predefined minimal required stepping frequency is reached or exceeded, the battery stays at equilibrium or fills. As long as the battery level is above 80 % (indicated by a line), the battery stays green. If the participants' stepping frequency falls below the predefined minimal required stepping frequency, the battery level decreases, and the battery turns orange (40 – 80 %) or red (below 40 %) indicating that the stepping frequency should be increased. On top of this fixed physical intensity, a variable amount of neurocognitive (game-) demands (e.g. game type, task complexity, predictability of required tasks) is applied. Since the physical intensity is kept constant, changes in the overall internal training load can mainly be attributed to these neurocognitive and motoric (game-) demands and, accordingly, the internal training load can be adjusted on basis of these game characteristics. Therefore, the neurocognitive demands of the exergame are individually adapted in order to ensure an appropriate total internal training load. The monitoring and adaption of the internal training load is based on predefined progression rules for adapting characteristics of external training load (section 2.7).

<u>Phase 2 – Guidance</u>

Phase 2 - Guidance aims to make use of the triggered neurophysiological mechanisms from phase 1 to specifically guide neuroplastic processes of the mainly impaired neurocognitive domain. Therefore, games focusing on the mainly impaired neurocognitive domain for the individual participant (e.g. amnestic single domain => learning and memory) are used. These games focus on cognitive and motoric demands, but not on physical intensity. The cognitive-motoric demands of the exergame (also called 'external load') are individually adapted in order to ensure an appropriate internal training load. The monitoring and adaption of the internal training load will be based on predefined progression rules for adapting characteristics of external training load (section 2.7).

<u>Phase 3 – Coherence</u>

Phase 3 - Coherence integrates HRV-BF training that includes breathing slowly and in a controlled manner and extending the exhalation phase. With this, we specifically activate the vagus nerve and promote the activation of the central autonomous networks in the brain that is important for self-regulation and the control of cognitive processes and helps to restore the balance of various physical systems. The unique combination of this biofeedback-guided breathing training with exergame training forms the core of our training concept. Through

physical and cognitive training, we bring various systems in the body out of balance. The breathing training aims to restore this balance and thus offer holistic training. Additionally, this also allows us to account for psychological factors, as patients with mNCD often exhibit depressive symptoms and anxiety, which are in turn important indicators for progression to dementia [14, 15].

HRV-BF training is a behavioral intervention aiming to increase cardiac autonomic control, enhance homeostatic regulation, and regulate emotional state [16-18]. It consists of a regular breathing practice at a specific frequency that is individually determined that produces high amplitude of heart rate variability (HRV), leading to increased cardiac autonomic control. Usually, this resonance breathing frequency is around 6 breaths/min [19]. An increased cardiac autonomic control increases vagal afferent transmission to the forebrain and activate and stimulate brain regions relevant for cognitive adaptations (such as the prefrontal cortex) [16, 18]. HRV-BF or paced breathing (at resonance frequency) is effective in improving cardiac autonomic control [18, 20], cognitive functioning (in particular executive functions) [21, 22], and emotional regulation [18, 22] (i.e., by decreasing symptoms of depression [18, 22, 23], anxiety [18, 23, 24], and stress [23, 24]) across different age groups and in clinical populations. The evidence for older adults (i.e. ≥ 60 years) or patients with cognitive impairments is sparse, but decreases in depression, anxiety, and increases in attentional performance (no sign. difference in executive functioning) have already been reported, suggesting that older adults may benefit from HRVBT much like the younger populations [25]. Moreover, there is evidence supporting a causal role of cardiac autonomic control in modulating plasma AD-related biomarkers [26].

In the ‘Brain-IT’ project, we did not have the resources to provide each participant with the technology to implement biofeedback throughout the intervention. However, “so far, no empirical evidence indicates that slow breathing practice with biofeedback offers superior outcomes in terms of vagally-mediated HRV or other health-related outcomes, compared to SPB without biofeedback.“ [27] Additionally, *“after initial training some people still achieve better results by following a heart monitor, while others do just as well doing paced breathing at their resonance frequency, once this frequency has been determined by biofeedback, following the second hand on a clock or counting seconds silently“* [4]. Therefore, for the sake of simplicity, made use of this transfer to resonance breathing. Before starting the training

intervention, the resonance frequency is determined according to the protocol of Lehrer et al. 2013 (i.e. visit 1 of their protocol) [28]. During the training intervention, HRV-BF training involves breathing for two minutes at a rhythm of 30 % inhale, 10 % hold, 50 % exhale, 10 % hold at the individually predetermined resonance frequency visualized on the screen of the exergame device (i.e. a sun is displayed within a landscape. When the sun gets bigger, the patients breath in. When the sun gets smaller, the patients breath out).

2.4.2 How to adapt the 'Brain-IT' training concept to other hardware and software solutions:

In general, the structure of the 'Brain-IT' training concept must remain the same (see Figure 1). To implement this, the following adaptations are required in each of the following phases of the training:

Phase 1 – Facilitation:

Various real-time feedback options can be provided in place of the battery number add-on to maintain a moderate level of physical intensity throughout the game. When implementing alternative options, the basic game design requirements (see Section 1.2 Overview of the Training) must be met. Otherwise, no adjustments to this phase are required.

Phase 2 – Guidance:

No adaptations are required for this phase.

Phase 3 – Coherence:

No adaptations are required for this phase. However, alternative (optimally gamified) visualizations can be used to guide participants' breathing patterns as long as the basic requirements for game design (see Section 1.2 Overview of the Training) are met.

2.5 Overview of Exergames and Trained Neurocognitive Domains

2.5.1 Implementation in the Project ‘Brain-IT’:

In this section, an overview of the currently available exergames on the ‘Senso (Flex)’ that we found suitable for implementation in the ‘Brain-IT’ project for the training of the neurocognitive domains of complex attention, learning and memory, executive function, and visuo-spatial skills that is provided. Depending on the complexity of the games as such, an earliest start and latest end were predefined that are considered in the progression rules (section 2.7).

Table 1: Overview of the currently available exergames on the ‘Senso (Flex)’ that we found suitable for implementation in the ‘Brain-IT’ project for the training of the neurocognitive domains of complex attention, learning and memory, executive function, and visuo-spatial skills

* = secondary classification of a game that focuses on more than one neurocognitive (sub)domains

color coding: black = existing games, green = new games or game elements that were developed in the ‘Brain-IT’ project

Training Focus	Neurocognitive Domain	Neurocognitive Subdomain	Exergames	Timeframe	
				earliest start	latest end
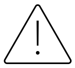	Complex Attention	Sustained Attention	‘Simple’	W 1	W 8
		Divided Attention	‘Divided’	W 2	W 10
		Selective Attention	‘Birds’	W 3	W 12
			‘Habitats’*	W 5	W 12
		Processing Speed	‘Simple’*	W 1	W 8
			‘Flexi’*	W 4	W 12
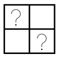	Learning & Memory AND Working Memory	Free Recall	‘Shopping Tour’*	W 1	W 12
		Serial Recall	‘Simon_numbered’	W 2	W 12
			‘Simon’	W 3	W 12
		Cued Recall	‘Steps’	W 4	W 12
		Recognition Memory	‘Shopping Tour’	W 1	W 12
		Semantic Memory	N/A	N/A	N/A
		Implicit Learning	N/A	N/A	N/A
		Working Memory	‘Nomis_numbered’	W 5	W 12
			‘Nomis’	W 6	W 12
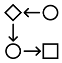	Executive Function	Planning	‘Targets’	W 1	W 12
			‘Tetris’*	W 6	W 12
		Decision Making	N/A	N/A	N/A
		Inhibition	‘Habitats’	W 5	W 12
		Flexibility	‘Flexi’	W 4	W 12
			‘Evolve’	W 2	W 12
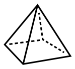	Visuo-spatial Skills	Visual Perception	‘Gears’*	W 1	W 12
			‘Tetris’*	W 6	W 12
			‘Targets’*	W 1	W 12
		Visuoconstructional Reasoning	‘Gears’	W 1	W 12
			‘Tetris’	W 6	W 12
		Perceptual-Motor Coordination	N/A	N/A	N/A

2.5.2 How to adapt the 'Brain-IT' training concept to other hardware and software solutions:

To adapt the 'Brain-IT' training concept to other software and hardware and software solutions, use Table 1 and fill in your existing exergames and/or develop new exergames in collaboration with neuropsychologist(s) and following these rules:

(1) Ideally, there should be at least one exergame available for each of the neurocognitive subdomains.

(2) Each exergame must be categorized into the primary neurocognitive subdomain being trained (and secondary subdomain in the case of a game that focuses on more than one neurocognitive (sub)domain). The categorization must be made by agreement between at least two experienced neuropsychologists to ensure the content validity of the exergames used to train each neurocognitive (sub)domain.

(3) For each game, an earliest and latest start time must be defined. The following steps should be followed, all in agreement with at least two experienced neuropsychologists, to ensure the content validity of the exergames used to train each neurocognitive (sub)domain.

I. For each neurocognitive domain, rank-order all available games according to their neurocognitive demands.

II. Allocate the least demanding game for each neurocognitive domain to start in the first week.

III. Allocate the remaining games consecutively according to their rank-order.

IV. In general, all games should be kept available until the end of the training to increase the available options of games throughout the training and in line with the concept of MYCHOICE. However, in case the neuropsychologists have good reasons for excluding games earlier in the training (e.g., introductory games that mainly fulfill the purpose of getting patients familiarized with the device, have limited options to increase neurocognitive demands, and where it is expected that they are not challenging enough even for the most impaired patients towords the end of the training), this can be defined accordingly.

2.6 Description of Specific Exergames

2.6.1 Implementation in the Project 'Brain-IT':

Table 2: Description of the currently available exergames on the 'Senso (Flex)' for the training focus on the neurocognitive domains of complex attention, learning and memory, executive function, and visuo-spatial skills

color coding: black = existing games, green = new games or game elements that were developed in the 'Brain-IT' project or related projects in our lab

Neurocognitive Domain	Exergames				
	Name	Main Neurocognitive Subdomain(s)	Description	Parameters to adapt Task Complexity	Feedback Mechanisms (provided after each response)
Complex Attention 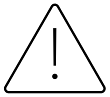	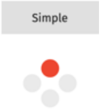	Sustained Attention, Processing Speed	In the game 'Simple', four circles are displayed in grey. As soon as one of the circles turns red, a step needs to be taken in the corresponding direction as fast as possible.	• internal progression algorithm that automatically adapts task difficulty based on game performance in real time<br>• game speed (interstimulus-interval)<br>• variance in interstimulus-interval<br>• response window<br>• predictability (predefined vs. random sequences)<br>• stepping direction(s)<br>• stepping frequency	<u>Positive feedback:</u> 'Positive' vibration (i.e. single short pulse), visual feedback (i.e. wiggling of target, and sound effect (i.e. "ringing bell")<br><u>Negative feedback:</u> 'Negative vibration' (i.e. multiple strong pulses).
	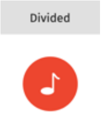	Divided Attention	In the game 'Divided', four circles are displayed in grey. As soon as one of the circles turns red, or an auditory cue is played (high tone = step forwards, low tone = step backwards), a step needs to be taken in the corresponding direction as fast as possible.	• internal progression algorithm that automatically adapts task difficulty based on game performance in real time<br>• game speed (interstimulus-interval)<br>• variance in interstimulus-interval<br>• response window<br>• predictability (predefined vs. random sequences)<br>• stepping direction(s)<br>• stepping frequency	<u>Positive feedback:</u> 'Positive' vibration (i.e. single short pulse), visual feedback (i.e. wiggling of target, and sound effect (i.e. "ringing bell").<br><u>Negative feedback:</u> 'Negative vibration' (i.e. multiple strong pulses).
	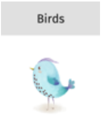	Selective Attention	In the game 'Birds' a feather is displayed in the middle of the screen. The participants' task is to match the feather with a bird and to return the feather to its birds by making a step into the corresponding direction.	• internal progression algorithm that automatically adapts task difficulty based on game performance in real time<br>• game speed (interstimulus-interval)<br>• variance in interstimulus-interval<br>• response window<br>• stepping direction(s)	<u>Positive feedback:</u> 'Positive' vibration (i.e. single short pulse), and sound effect (i.e. bird chirping).<br><u>Negative feedback:</u> 'Negative vibration' (i.e. multiple strong pulses) and

				• predictability (predefined vs. random sequences)<br>• stepping frequency	sound effect (i.e. muffled sound effect).
Learning and Memory & Working Memory (EF)<br>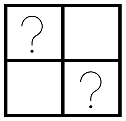	Simon_numbered<br>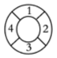	Serial Recall	In the game 'Simon_numbered', a stepping sequence (i.e. indicated by a concurrent lighting up of a sequence of sections with different numbers of a circle and a corresponding sound) has to be memorized and repeated by stepping into the corresponding direction.	• sequence length<br>• stepping direction(s)<br>• stepping frequency	Positive feedback:<br>'Positive' vibration (i.e. single short pulse), visual feedback (i.e. lighting up of target, and sound effect (i.e. single tone corresponding to color).<br><br>Negative feedback:<br>'Negative vibration' (i.e. multiple strong pulses) and sound effect (i.e. muffled sound effect).
	Simon<br>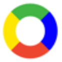	Serial Recall	In the game 'Simon', a stepping sequence (i.e. indicated by a concurrent lighting up of a sequence of sections with different colors of a circle and a corresponding sound) has to be memorized and repeated by stepping into the corresponding direction.	• internal progression algorithm that automatically adapts task difficulty based on game performance in real time<br>• sequence length<br>• stepping direction(s)<br>• stepping frequency	Positive feedback:<br>'Positive' vibration (i.e. single short pulse), visual feedback (i.e. lighting up of target, and sound effect (i.e. single tone corresponding to color).<br><br>Negative feedback:<br>'Negative vibration' (i.e. multiple strong pulses) and sound effect (i.e. muffled sound effect).
	Steps<br>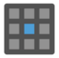	Cued Recall	In the game 'Steps', a stepping sequence (i.e. indicated by a concurrent lighting up of a sequence of sections with different numbers of a square with 9 fields) has to be memorized and repeated by stepping into the corresponding direction at the rhytm of the beat (i.e. indicated by a metronome).	• number of steps per level<br>• maximal number of trials per stepping sequence<br>• start level	Positive feedback:<br>'Positive' vibration (i.e. single short pulse), visual feedback (i.e. lighting up of target (in green), and sound effect (i.e. single tone corresponding to a number and ascending tone sequence).<br><br>Negative feedback:<br>visual feedback (i.e. lighting up of target (in orange/red), and sound effect (i.e. descending tone sequence).

	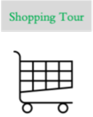	Free Recall, Recognition Memory	In the game 'Shopping Tour, at first, a shopping list is displayed in the center of the screen for the duration of encoding phase. Second, the shopping list will disappear and one item after another will appear on the screen. The users' task is to gather all items (type and quantity) of the shopping list by stepping to the right (i.e. to put the object into the shopping cart) or to the left (i.e. not to buy the product). After each response, a feedback is provided.	• number of items on the list<br>• number of items to be purchased<br>• probability of presented items to be purchased or not (in percent)<br>• probability that items need to be purchased multiple times (in percent)	Positive feedback:<br>Arrow lights up in green light, 'positive' (i.e. single short pulse) vibration and sound effect (i.e. "ringing bell"), cross out items on shopping list and throw out list when successfully completed<br><br>Negative feedback:<br>Arrow lights up in red light, 'negative vibration' (i.e. multiple strong pulses), and sound effect (i.e. muffled sound effect).
	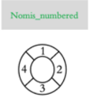	Working Memory	In the game 'Nomis_numbered', a stepping sequence (i.e. indicated by a concurrent lighting up of a sequence of sections with different numbers of a circle and a corresponding sound) has to be memorized and repeated backwards by stepping into the corresponding direction.	• sequence length<br>• stepping direction(s)<br>• stepping frequency	Positive feedback:<br>'Positive' vibration (i.e. single short pulse), visual feedback (i.e. lighting up of target, and sound effect (i.e. single tone corresponding to color).<br><br>Negative feedback:<br>'Negative vibration' (i.e. multiple strong pulses) and sound effect (i.e. muffled sound effect).
	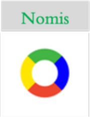	Working Memory	In the game 'Nomis' (Simon backwards), a stepping sequence (i.e. indicated by a concurrent lighting up of a sequence of sections with different colors of a circle and a corresponding sound) has to be memorized and repeated backwards by stepping into the corresponding direction.	• sequence length<br>• stepping direction(s)<br>• stepping frequency	Positive feedback:<br>'Positive' vibration (i.e. single short pulse), visual feedback (i.e. lighting up of target, and sound effect (i.e. single tone corresponding to color).<br><br>Negative feedback:<br>'Negative vibration' (i.e. multiple strong pulses) and sound effect (i.e. muffled sound effect).
Executive Function<br>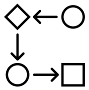	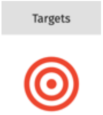	Planning	The game 'Targets' requires the participant to hit incoming red balls in the middle of the target by stepping into the corresponding direction.	• internal progression algorithm that automatically adapts task difficulty based on game performance in real time<br>• game speed (speed multiplier)<br>• stepping direction(s)<br>• stepping frequency	Positive feedback:<br>'Positive' vibration (i.e. single short pulse), visual feedback (i.e. visual impulse of selected target, and sound effect (i.e. "ringing bell").<br><br>Negative feedback:

					‘Negative vibration’ (i.e. multiple strong pulses) and sound effect (i.e. muffled sound effect).
	Habitats 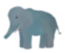	Inhibition, Selective Attention	In the game ‘Habitats’, animals move across the four landscapes in the picture. If an animal does not appear in its familiar surroundings, a step needs to be taken in this direction. However, animals shouldn’t be disturbed in their natural habitat.	• internal progression algorithm that automatically adapts task difficulty based on game performance in real time<br>• game speed (interstimulus-interval)<br>• variance in interstimulus-interval<br>• task complexity (including inhibition tasks or not)<br>• stepping direction(s)<br>• stepping frequency	<u>Positive feedback</u>:<br>‘Positive’ vibration (i.e. single short pulse), visual feedback (i.e. wiggling of target, and sound effect (i.e. animal sounds).<br><br><u>Negative feedback:</u><br>‘Negative vibration’ (i.e. multiple strong pulses), sound effect (i.e. muffled sound effect), and visual animation (i.e. animal with speech bubble displaying “Hey!”)
	Flexi 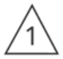	Flexibility, Processing Speed	The game ‘Flexi’ consists of two parts:<br><u>Part A:</u> Requires participants to make a step in the direction of the next higher number, starting from the number displayer in the center.<br><u>Part B:</u> In addition to the task of Part A, a figure appears around the number. It is necessary to make a step in the direction of the next higher number with the opposite pattern.	• task complexity (Part A/B)	<u>Positive feedback:</u><br>‘Positive’ vibration (i.e. single short pulse), and sound effect (i.e. “ringing bell”).<br><br><u>Negative feedback:</u><br>‘Negative vibration’ (i.e. multiple strong pulses).
	Evolve 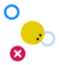	Flexibility	The task of the game ‘Evolve’ is to catch targets (blue balls) while avoiding hitting obstacles (red crosses) by controlling an avatar through weight shifting.	• progression that automatically adapts task difficulty based on game performance in real time<br>• game speed (interstimulus-interval) of targets<br>• movement speed of targets<br>• game speed (interstimulus-interval) of obstacles<br>• movement speed of obstacles	<u>Positive feedback:</u><br>‘Positive’ vibration (i.e. single short pulse), and sound effect (i.e. high “blubb” sound).<br><br><u>Negative feedback:</u><br>‘Negative vibration’ (i.e. multiple strong pulses) and sound effect (i.e. low “blubb” sound).
Visuo-Spatial Skills 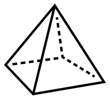	Gears 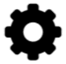	Visuoconstructional Reasoning, Planning	‘Gears’ requires the participant to select the correct trace for the corresponding gear wheel of a displayed train.	• complexity of the gear wheels (3 selectable levels)<br>• response window<br>• stepping direction(s)<br>• predictability (predefined vs. random sequences)	<u>Positive feedback:</u><br>‘Positive’ vibration (i.e. single short pulse), visual feedback (i.e. visual impulse of selected target, and sound effect (i.e. “ringing bell”).<br><br><u>Negative feedback:</u>

				• animated (rotating wheels) wheels vs. wheels in fixed position<br>• stepping frequency	'Negative vibration' (i.e. multiple strong pulses) and sound effect (i.e. muffled sound effect).
	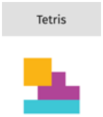	Visuoconstructional Reasoning, Planning	'Tetris' requires participants to rotate and move two-dimensional polygons (with varying shapes and colors) dropping one-by-one from top to the bottom. The aim of the game is to arrange complete rows of blocks to form solid horizontal lines, in order to let these lines disappear.	• internal progression algorithm that automatically adapts task difficulty based on game performance in real time<br>• game speed (speed multiplier)<br>• stepping frequency	Positive feedback:<br>'Positive' vibration (i.e. multiple short pulses) for a full row, visual feedback (i.e. full row disappears), and sound effect (i.e. "ringing bell").<br><br>Negative feedback:<br>none

2.6.2 How to adapt the ‘Brain-IT’ training concept to other hardware and software solutions:

In Table 2, provide a description of each of the exergames you used to deliver the 'Brain-IT' training.

2.7 Progression Rules for Monitoring Internal Load and Adapting External Loads

2.7.1 Phase 1 – Facilitation

Implementation in the Project ‘Brain-IT’:

As described above, the internal training load is subdivided into the physical exercise intensity of the stepping task and the neurocognitive and motoric (game-) demands of the games in phase 1. Additionally, the level of stability support (holding on to a handrail or similar with both hands, one hand, only two fingers or no stability support) is individually determined to provide a challenging but safe condition. The stepping frequency of the stepping tasks is predetermined for each participant with the aim to reach a moderate level of physical intensity (i.e. ranging between 40 and 59 % heart rate reserve (HRR) [13]). To avoid overload, the participants are introduced stepwise; first, the stepping frequency is determined while the level of neurocognitive demand is held low. Afterwards, the total level of internal training load is monitored and continuously adapted.

Phase 1a - Determination of minimal stepping frequency:

All participants start with a stepping frequency of 100 steps/min and at Level 1 of game demands in the first training session (see section 7). The target physical exercise intensity is determined based on the target heart rate (HR) that is calculated using the Karvonen method with a target intensity of 40 % HRR: $HR_{target} = (HR_{max} – HR_{rest}) \cdot 0.40 + HR_{rest}$ [29, 30]. For this calculation the age-predicted maximal heart rate: $HR_{max} = 208 – 0.7 \cdot age$ and HR_{rest} measured at the pre-measurements is used. The stepping frequency is then increased by 5 steps/min at each training session (to a maximum of 140 steps/min) until the minimal level of physical exercise intensity is reached. The evaluated stepping frequency is then considered as a fixed component of the overall external load. In all subsequent training session, this fixed physical exercise intensity is kept constant and the focus shifts on monitoring and adapting the total internal training load.

Phase 1b – Monitoring and adaptation of total internal training load:

Since the physical intensity in phase 2 is held constant, changes in the overall internal training load can mainly be attributed to the variable level of neurocognitive demand. The level of neurocognitive demand is standardized according to predefined game levels (see section 7). Phase 2 continues with game level 1, until a plateau in performance is reached. A plateau in performance is read out visually guided by the following predefined criteria: (1) a performance increase of less than or equal to 5 % compared to the previous exergame session while (2) there was an increase in performance from session so session over at least the previous three training sessions. The specific performance outcomes for each exergame to take into consideration are underlined in table 3. In case a precision outcome is available (i.e. for the game 'Targets'; precision = number of hits / (number of hits + number of missed targets)), the criterion to progress to the next higher level is to achieve a precision of at least 90 %. Each time a plateau in performance is reached, the game level is increased by one level. If the participants wishes to have the task demands increased or the staff supervising the participants recognize that an increase in the task demands is feasible, they have the option to override these progression criteria and increase the game level by one (or more) level(s). Additionally, the level of stability support (holding on to a handrail or similar with both hands, one hand, only two fingers or no stability support) is individually determined to reach a challenging but safe condition. Depending on the complexity of the games as such, the earliest start and latest end that were predefined in section 2 that are additionally considered when planning the training sessions.

<u>How to adapt the 'Brain-IT' training concept to alternative exergame devices:</u>

In general, the progression rules for monitoring the internal training load and adapting the external training loads must remain the same as implemented in the 'Brain-IT' project. To adapt the 'Brain-IT' training to other software and hardware and software solutions, use all available exergames filled in Table 1 and fill in Table 3 for each of these games. The game levels and the parameters chosen to reach these levels must be defined by agreement between at least two experienced neuropsychologists, taking into account the following key points:

- Level 1 = Introductory level. Even most impaired patients should be able to play the game while performing the additional stepping task at the first trial without problems to ensure that no overload occurs.
- Level 10 = "healthy functioning" level. The neurocognitive demands, while performing the additional step task, are expected to be challenging but doable for an average healthy older adult.
- The remaining levels are defined to increase neurocognitive demands consecutively and regularly from level to level. This should again be done by agreement between at least two experienced neuropsychologists. It is recommended to consider Gentile's Taxonomy for Motor Learning [31], Neuroplasticity Principles [32], Motor Learning Principles [33], and Training Principles [34, 35] in this regard.

For each game, at least one game metric needs to be chosen or developed that provides a valid and reliable measure for game performance and is sensitive to changes in game performance over time.

In case a scientifically validated progression algorithm that is based on game metric that provides a valid and reliable measure for game performance and are sensitive to changes in game performance over time, this option can be considered instead of the predefined levels. However, it must be ensured, that the patients are not overloaded in the initial training sessions.

2.7.2 Phase 2 – Guidance

<u>Implementation in the Project 'Brain-IT':</u>

In phase 2, the mainly impaired neurocognitive domain is trained. Therefore, the focus of monitoring and adapting the task demands is on neurocognitive demands (i.e. motor- and cognitive demands that are linked because both change as a function of game complexity). The level of neurocognitive demand is standardized according to predefined game levels (see section 8) for game levels one to nine. The final game level (i.e. Level 10+) is based on an adaptive mode (i.e. internal progression algorithm provided by the Dividat) that automatically adapts task difficulty based on game performance in real time and aims to adapt the game demands in order to provide an optimal challenge.

All participants start with level 1. Each time a plateau in performance is reached, the game level is increase by one level. A plateau in performance is read out visually guided by the following predefined criteria: (1) a performance increase of less than or equal to 5 % compared to the previous exergame session while (2) there was an increase in performance from session so session over at least the previous three training sessions. The specific performance outcomes for each exergame to take into consideration are underlined in table 4. In case a precision outcome is available (i.e. for the games ‘Shopping Tour’ and ‘Targets’ (precision = number of hits / (number of hits + number of missed targets))), the criterion to progress to the next higher level is to achieve a precision of at least 90 %. If the participants wish to have the task demands increased or the staff supervising the participants recognize that an increase in the task demands is feasible, they have the option to override these progression criteria and increase the game level by one (or more) level(s). Additionally, the level of stability support (holding on to a handrail or similar with both hands, one hand, only two fingers or no stability support) is individually determined to reach a challenging but safe condition. Depending on the complexity of the games as such, the earliest start and latest end that were predefined in section 2 that are additionally considered when planning the training sessions.

<u>How to adapt the ‘Brain-IT’ training concept to other hardware and software solutions:</u>

In general, the progression rules for monitoring the internal training load and adapting the external training loads must remain the same as implemented in the 'Brain-IT' project. To adapt the 'Brain-IT' training to other software and hardware and software solutions, use all available exergames filled in Table 1 and fill in Table 4 for each of these games. The game levels and the parameters chosen to reach these levels must be defined by agreement between at least two experienced neuropsychologists, taking into account the following key points:

- Level 1 = Introductory level. Even most impaired patients should be able to play the game at the first trial without problems to ensure that no overload occurs.
- Level 10 = "healthy functioning" level. The neurocognitive demands are expected to be challenging but doable for an average healthy older adult.
- The remaining levels are defined to increase neurocognitive demands consecutively and regularly from level to level. This should again be done by agreement between at

least two experienced neuropsychologists. It is recommended to consider Gentile's Taxonomy for Motor Learning [31], Neuroplasticity Principles [32], Motor Learning Principles [33], and Training Principles [34, 35] in this regard.

For each game, at least one game metric needs to be chosen or developed that provides a valid and reliable measure for game performance and is sensitive to changes in game performance over time.

In case a scientifically validated progression algorithm that is based on game metric that provides a valid and reliable measure for game performance and are sensitive to changes in game performance over time, this option can be considered instead of the predefined levels. However, it must be ensured, that the patients are not overloaded in the initial training sessions.

2.8 The concept of MYCHOICE to ensure sufficient variability

2.8.1 Implementation in the Project 'Brain-IT':

The concept of MYCHOICE describes a self-determined choice of exergames within groups of games for cognitive domains so that the preferences of each participant can be taken into account while the time spent at training each neurocognitive domain is still standardized within participants with the same training focus (i.e. predetermined according to the deficit-oriented focus on the neurocognitive domains as described in section 2). The advantage of this concept is that it promotes self-efficacy, which facilitates training motivation [36]. According to the Optimizing Performance through Intrinsic Motivation and Attention for Learning (OPTIMAL) theory of motor learning, this is expected to enhance performance expectancies which – accompanied with these autonomy-supportive conditions - *"contribute to efficient goal-action coupling by preparing the motor system for task execution"* [37]. This is further proposed *"to facilitate the development of functional connectivity across brain regions, and structural neural connections more locally, that support effective and efficient motor performance and learning"* [37, 38]. With this regard, the exergames were grouped into mainly trained neurocognitive domains of learning and memory, executive function, complex attention, visuo-spatial skills (see table 1) and each participant gets to choose which game within these groups he prefers to play. Optimally, the participant would get the option to choose between different games on the screen before starting each training session. Since

this is not (yet) implemented into the Dividat user interface, alternatively, the research team consecutively plans the training session for each participant based on the participant's preferences.

Depending on the complexity of the games as such, the earliest start and latest end that are predefined in section 2 that have to be considered when planning the training sessions. Therefore, the range for self-determined choices is limited at the start of the training with the aim to provide a certain routine until the participants have familiarized themselves with the game scenarios and are prepared to learn new games step-by-step. Over the course of the training intervention, the number of options to choose from will steadily increase, giving the participants and the research team the opportunity to plan the training sessions according to the individuals' preferences.

2.8.2 How to adapt the 'Brain-IT' training concept to other hardware and software solutions:

Ideally, no changes to this concept are required if the exergame software allows for the grouping of alternative exergames to train the same neurocognitive (sub)domain and these can be displayed on the interface for selection by the participants before starting or during the training. If this option is not available, the person supervising the participants should consecutively plan the training session for each participant based on the participant's preferences.

2.9 Game Levels for Phase 1 – Facilitation

Table 3: Game Levels for each Game for Phase 1 – Facilitation

Game	Task Demands													Performance Measures
	Parameter(s)		Conditions & Settings											
			Level 1	Level 2	Level 3	Level 4	Level 5	Level 6	Level 7	Level 8	Level 9	Level 10	
Simple 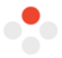	game speed (interstimulus-interval)		= ½ RW	= ½ RW	= ½ RW	= ½ RW	= ½ RW	= ½ RW	= ½ RW	= ½ RW	= ½ RW	= ½ RW	<u>mean reaction time</u><br>game score<br>point rate
	response window (RW)		10000 ms	8000 ms	6000 ms	5500 ms	5000 ms	4500 ms	4000 ms	3500 ms	3000 ms	2500 ms	
	predictability (order/time interval)		random	random	random	random	random	random	random	random	random	random	
	stepping direction(s)	↑	90 %	80 %	70 %	60 %	50 %	45 %	40 %	35 %	30 %	25 %	
		→	5 %	10 %	15 %	20 %	22.5 %	22.5 %	25 %	25 %	25 %	25 %	
		←	5 %	10 %	15 %	20 %	22.5 %	22.5 %	25 %	25 %	25 %	25 %	
		↓	0 %	0 %	0 %	0 %	5 %	10 %	10 %	15 %	20 %	25 %	
Divided 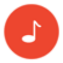	game speed (interstimulus-interval)		= ½ RW	= ½ RW	= ½ RW	= ½ RW	= ½ RW	= ½ RW	= ½ RW	= ½ RW	= ½ RW	= ½ RW	<u>mean reaction time</u><br>game score<br>point rate
	response window (RW)		12000 ms	10000 ms	8000 ms	6000 ms	5500 ms	5000 ms	4500 ms	4000 ms	3500 ms	3000 ms	
	predictability (order/time interval)		random	random	random	random	random	random	random	random	random	random	
	stepping direction(s)	↑	80 %	70 %	60 %	55 %	50 %	45 %	40 %	35 %	30 %	25 %	
		→	10 %	15 %	20 %	20 %	22.5 %	22.5 %	25 %	25 %	25 %	25 %	
		←	10 %	15 %	20 %	20 %	22.5 %	22.5 %	25 %	25 %	25 %	25 %	
		↓	0 %	0 %	0 %	5 %	5 %	10 %	10 %	15 %	20 %	25 %	
Birds 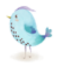	game speed (interstimulus-interval)		= ½ RW	= ½ RW	= ½ RW	= ½ RW	= ½ RW	= ½ RW	= ½ RW	= ½ RW	= ½ RW	= ½ RW	<u>mean reaction time</u><br>game score<br>point rate
	response window (RW)		10000 ms	8000 ms	6000 ms	5500 ms	5000 ms	4500 ms	4000 ms	3500 ms	3000 ms	2500 ms	
	predictability (order/time interval)		random	random	random	random	random	random	random	random	random	random	
	stepping direction(s)	↑	80 %	70 %	60 %	55 %	50 %	45 %	40 %	35 %	30 %	25 %	
		→	10 %	15 %	20 %	20 %	22.5 %	22.5 %	25 %	25 %	25 %	25 %	
		←	10 %	15 %	20 %	20 %	22.5 %	22.5 %	25 %	25 %	25 %	25 %	
		↓	0 %	0 %	0 %	5 %	5 %	10 %	10 %	15 %	20 %	25 %	

Simon_numbered	sequence length		2	3	3	3	4	4	5	5	6	6	mean reaction time<br>game score<br>point rate
	stepping direction(s)	↑	50 %	50 %	35 %	25 %	35 %	25 %	35 %	25 %	35 %	25 %	
		→	25 %	25 %	25 %	25 %	25 %	25 %	25 %	25 %	25 %	25 %	
		←	25 %	25 %	25 %	25 %	25 %	25 %	25 %	25 %	25 %	25 %	
		↓	0 %	0 %	15 %	25 %	15 %	25 %	15 %	25 %	15 %	25 %	
Simon	sequence length		2	3	3	3	4	4	5	5	6	6	mean reaction time<br>game score<br>point rate
	stepping direction(s)	↑	50 %	50 %	35 %	25 %	35 %	25 %	35 %	25 %	35 %	25 %	
		→	25 %	25 %	25 %	25 %	25 %	25 %	25 %	25 %	25 %	25 %	
		←	25 %	25 %	25 %	25 %	25 %	25 %	25 %	25 %	25 %	25 %	
		↓	0 %	0 %	15 %	25 %	15 %	25 %	15 %	25 %	15 %	25 %	
Shopping Tour	number of items on the list		2	3	3	4	4	5	5	6	6	7	mean reaction time<br>number of items collected<br>number of mistakes<br>precision
	duration of encoding phase		10 s	8 s	6 s	8 s	6 s	7 s	5 s	9 s	6 s	7 s	
	bulking probability		0 %	80 %	60 %	80 %	60 %	60 %	40 %	50 %	40 %	50 %	
	probability of presented items to be purchased or not (in percent)		80 %	70 %	60 %	55 %	50 %	45 %	40 %	35 %	30 %	25 %	
Nomis_numbered	sequence length		2	3	3	3	4	4	4	5	5	6	mean reaction time<br>game score<br>point rate
	stepping direction(s)	↑	50 %	50 %	35 %	25 %	35 %	25 %	35 %	25 %	35 %	25 %	
		→	25 %	25 %	25 %	25 %	25 %	25 %	25 %	25 %	25 %	25 %	
		←	25 %	25 %	25 %	25 %	25 %	25 %	25 %	25 %	25 %	25 %	
		↓	0 %	0 %	15 %	25 %	15 %	25 %	15 %	25 %	15 %	25 %	
Nomis	sequence length		2	3	3	3	4	4	4	5	5	6	mean reaction time<br>game score<br>point rate
	stepping direction(s)	↑	50 %	50 %	35 %	25 %	35 %	25 %	35 %	25 %	35 %	25 %	
		→	25 %	25 %	25 %	25 %	25 %	25 %	25 %	25 %	25 %	25 %	
		←	25 %	25 %	25 %	25 %	25 %	25 %	25 %	25 %	25 %	25 %	
		↓	0 %	0 %	15 %	25 %	15 %	25 %	15 %	25 %	15 %	25 %	
Targets	game speed (speed multiplier)		0.2	0.25	0.3	0.35	0.4	0.45	0.5	0.55	0.6	0.65	game score<br>point rate<br>number of hits<br>number of missed targets
	stepping direction(s)	↑	90 %	80 %	70 %	60 %	50 %	45 %	40 %	35 %	30 %	25 %	
		→	5 %	10 %	15 %	20 %	22.5 %	22.5 %	25 %	25 %	25 %	25 %	
		←	5 %	10 %	15 %	20 %	22.5 %	22.5 %	25 %	25 %	25 %	25 %	
		↓	0 %	0 %	0 %	0 %	5 %	10 %	10 %	15 %	20 %	25 %	
Habitats	game speed (interstimulus-interval)		10000 ms	8000 ms	6000 ms	5500 ms	5000 ms	4500 ms	4000 ms	3500 ms	3000 ms	2500 ms	mean reaction time<br>game score<br>point rate
	response window (RW)		constant	constant	constant	constant	constant	constant	constant	constant	constant	constant	
	task complexity (including inhibition task = yes/no)		no	no	no	yes	yes	yes	yes	yes	yes	yes	
	stepping direction(s)	↑	80 %	70 %	60 %	55 %	50 %	45 %	40 %	35 %	30 %	25 %	
		→	10 %	15 %	20 %	20 %	22.5 %	22.5 %	25 %	25 %	25 %	25 %	
		←	10 %	15 %	20 %	20 %	22.5 %	22.5 %	25 %	25 %	25 %	25 %	
		↓	0 %	0 %	0 %	5 %	5 %	10 %	10 %	15 %	20 %	25 %	

Gears	response window (RW)		12000 ms	10000 ms	8000 ms	8000 ms	8000 ms	6000 ms	6000 ms	5000 ms	5000 ms	5000 ms	<u>mean reaction time</u><br>game score<br>point rate
	complexity of the gear wheels (out of 3 levels)		Level 1	Level 1	Level 1	Level 1&2	Level 1&2	Level 1&2	Level 2	Level 2	Level 2&3	Level 3	
	animated (rotating wheels) wheels vs. wheels in fixed position		fixed	fixed	fixed	fixed	animated	animated	animated	animated	animated	animated	
	predictability (order/time interval)		random	random	random	random	random	random	random	random	random	random	
	stepping direction(s)	↑	80 %	60 %	40 %	35 %	35 %	30 %	30 %	25 %	25 %	25 %	
		→	10 %	20 %	25 %	25 %	25 %	25 %	25 %	25 %	25 %	25 %	
		←	10 %	20 %	25 %	25 %	25 %	25 %	25 %	25 %	25 %	25 %	
		↓	0 %	0 %	10 %	15 %	15 %	20 %	20 %	25 %	25 %	25 %	
Tetris	game speed (speed multiplier)		0.6	0.8	1	1.2	1.3	1.4	1.5	1.6	1.7	1.8	<u>game score</u>

2.10 Game Levels for Phase 2 – Guidance

Table 4: Game Levels for each Game for Phase 2 – Guidance

Game	Task Demands												Performance Measures
	Parameter(s)		Conditions & Settings										
			Level 1	Level 2	Level 3	Level 4	Level 5	Level 6	Level 7	Level 8	Level 9	Level 10(+)	
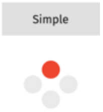	game speed (interstimulus-interval)		= ½ RW	= ½ RW	= ½ RW	= ½ RW	= ½ RW	= ½ RW	= ½ RW	= ½ RW	= ½ RW	= ½ RW	<u>mean reaction time</u><br>game score<br>point rate
	response window (RW)		6000 ms	5000 ms	4500 ms	4000 ms	3750 ms	3500 ms	3250 ms	3000 ms	2750 ms	adaptive; start level: 2500 ms	
	predictability (order/time interval)		random	random	random	random	random	random	random	random	random	random	
	stepping direction(s)	↑	90 %	80 %	70 %	60 %	50 %	45 %	40 %	35 %	30 %	25 %	
		→	5 %	10 %	15 %	20 %	22.5 %	22.5 %	25 %	25 %	25 %	25 %	
		←	5 %	10 %	15 %	20 %	22.5 %	22.5 %	25 %	25 %	25 %	25 %	
		↓	0 %	0 %	0 %	0 %	5 %	10 %	10 %	15 %	20 %	25 %	
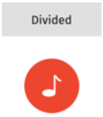	game speed (interstimulus-interval)		= ½ RW	= ½ RW	= ½ RW	= ½ RW	= ½ RW	= ½ RW	= ½ RW	= ½ RW	= ½ RW	= ½ RW	<u>mean reaction time</u><br>game score<br>point rate
	response window (RW)		5000 ms	4500 ms	4000 ms	3750 ms	3500 ms	3250 ms	3000 ms	2750 ms	2500 ms	adaptive; start level: 2250 ms	
	predictability (order/time interval)		random	random	random	random	random	random	random	random	random	random	
	stepping direction(s)	↑	80 %	70 %	60 %	55 %	50 %	45 %	40 %	35 %	30 %	25 %	
		→	10 %	15 %	20 %	20 %	22.5 %	22.5 %	25 %	25 %	25 %	25 %	
		←	10 %	15 %	20 %	20 %	22.5 %	22.5 %	25 %	25 %	25 %	25 %	
		↓	0 %	0 %	0 %	5 %	5 %	10 %	10 %	15 %	20 %	25 %	
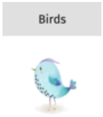	game speed (interstimulus-interval)		= ½ RW	= ½ RW	= ½ RW	= ½ RW	= ½ RW	= ½ RW	= ½ RW	= ½ RW	= ½ RW	= ½ RW	<u>mean reaction time</u><br>game score<br>point rate
	response window (RW)		8000 ms	6000 ms	5500 ms	5000 ms	4500 ms	4000 ms	3500 ms	3000 ms	2500 ms	adaptive; start level: 2000 ms	
	predictability (order/time interval)		random	random	random	random	random	random	random	random	random	random	
	stepping direction(s)	↑	80 %	70 %	60 %	55 %	50 %	45 %	40 %	35 %	30 %	25 %	
		→	10 %	15 %	20 %	20 %	22.5 %	22.5 %	25 %	25 %	25 %	25 %	
		←	10 %	15 %	20 %	20 %	22.5 %	22.5 %	25 %	25 %	25 %	25 %	
		↓	0 %	0 %	0 %	5 %	5 %	10 %	10 %	15 %	20 %	25 %	

Simon_numbered	sequence length		2	3	3	4	4	5	5	6	6	7	mean reaction time<br>game score<br>point rate
	stepping direction(s)	↑	50 %	50 %	35 %	35 %	25 %	35 %	25 %	35 %	25 %	25 %	
		→	25 %	25 %	25 %	25 %	25 %	25 %	25 %	25 %	25 %	25 %	
		←	25 %	25 %	25 %	25 %	25 %	25 %	25 %	25 %	25 %	25 %	
		↓	0 %	0 %	15 %	15 %	25 %	15 %	25 %	15 %	25 %	25 %	
Simon	sequence length		2	3	3	4	4	5	5	6	6	7	mean reaction time<br>game score<br>point rate
	stepping direction(s)	↑	50 %	50 %	35 %	35 %	25 %	35 %	25 %	35 %	25 %	25 %	
		→	25 %	25 %	25 %	25 %	25 %	25 %	25 %	25 %	25 %	25 %	
		←	25 %	25 %	25 %	25 %	25 %	25 %	25 %	25 %	25 %	25 %	
		↓	0 %	0 %	15 %	15 %	25 %	15 %	25 %	15 %	25 %	25 %	
Steps	progression rule defined by the game itself		This game was designed to include over 100 game levels considering a progression in motor load (i.e. execution speed (i.e. stepping frequency (beats per minute) and pattern complexity), and cognitive load (i.e. pattern length) that are described by Giannouli et al. 2020 [39]. All participants will start at level 1 and each training session will start at the final level of the previous training session.										game score<br>point rate<br>number of hits<br>number of missed targets<br>accuracy
Shopping Tour	number of items on the list		2	3	3	4	4	5	5	6	6	7	mean reaction time<br>number of items collected<br>number of mistakes<br>precision
	duration of encoding phase		10 s	8 s	6 s	8 s	6 s	7 s	5 s	9 s	6 s	7 s	
	bulking probability		0 %	80 %	60 %	80 %	60 %	60 %	40 %	50 %	40 %	50 %	
	probability of presented items to be purchased or not (in percent)		80 %	70 %	60 %	55 %	50 %	45 %	40 %	35 %	30 %	25 %	
Nomis_numbered	sequence length		2	3	3	3	4	4	5	5	6	6	mean reaction time<br>game score<br>point rate
	stepping direction(s)	↑	50 %	50 %	35 %	25 %	35 %	25 %	35 %	25 %	35 %	25 %	
		→	25 %	25 %	25 %	25 %	25 %	25 %	25 %	25 %	25 %	25 %	
		←	25 %	25 %	25 %	25 %	25 %	25 %	25 %	25 %	25 %	25 %	
		↓	0 %	0 %	15 %	25 %	15 %	25 %	15 %	25 %	15 %	25 %	
Nomis	sequence length		2	3	3	3	4	4	5	5	6	6	mean reaction time<br>game score<br>point rate
	stepping direction(s)	↑	50 %	50 %	35 %	25 %	35 %	25 %	35 %	25 %	35 %	25 %	
		→	25 %	25 %	25 %	25 %	25 %	25 %	25 %	25 %	25 %	25 %	
		←	25 %	25 %	25 %	25 %	25 %	25 %	25 %	25 %	25 %	25 %	
		↓	0 %	0 %	15 %	25 %	15 %	25 %	15 %	25 %	15 %	25 %	

Targets	game speed (speed multiplier)		0.3	0.4	0.5	0.55	0.6	0.65	0.7	0.75	0.8	adaptive; start level: 0.8	game score<br>point rate<br>number of hits<br>number of missed targets
	stepping direction(s)	↑	90 %	80 %	70 %	60 %	50 %	45 %	40 %	35 %	30 %	25 %	
		→	5 %	10 %	15 %	20 %	22.5 %	22.5 %	25 %	25 %	25 %	25 %	
		←	5 %	10 %	15 %	20 %	22.5 %	22.5 %	25 %	25 %	25 %	25 %	
		↓	0 %	0 %	0 %	5 %	5 %	10 %	10 %	15 %	20 %	25 %	
Habitats	game speed (interstimulus-interval)		8000 ms	6000 ms	5000 ms	4500 ms	4000 ms	3500 ms	3250 ms	3000 ms	2750 ms	adaptive; start level: 2500 ms	mean reaction time<br>game score<br>point rate
	response window (RW)		constant	constant	constant	constant	constant	constant	constant	constant	constant	constant	
	task complexity (including inhibition task = yes/no)		no	no	no	yes	yes	yes	yes	yes	yes	yes	
	stepping direction(s)	↑	80 %	70 %	60 %	55 %	50 %	45 %	40 %	35 %	30 %	25 %	
		→	10 %	15 %	20 %	20 %	22.5 %	22.5 %	25 %	25 %	25 %	25 %	
		←	10 %	15 %	20 %	20 %	22.5 %	22.5 %	25 %	25 %	25 %	25 %	
		↓	0 %	0 %	0 %	5 %	5 %	10 %	10 %	15 %	20 %	25 %	
Flexi	task complexity		Part A	Part A & B									mean reaction time<br>game score
Evolve	game speed (interstimulus-interval) of targets		10000 ms	7500 ms	5000 ms	5000 ms	4000 ms	4000 ms	4000 ms	4000 ms	4000 ms	4000 ms	catches (Level 1-3)<br>collisions<br>precision (Level 4+)<br>points
	movement speed of targets		0	0	0	0.2	0.4	0.6	0.7	0.8	0.9	1	
	game speed (interstimulus-interval) of obstacles		0	0	0	10000 ms	8000 ms	6000 ms	4000 ms	3000 ms	2000 ms	1000 ms	
	movement speed of obstacles		0	0	0	0.2	0.4	0.6	0.7	0.8	0.9	1	
Gears	response window (RW)		10000 ms	8000 ms	6000 ms	6000 ms	6000 ms	5000 ms	5000 ms	4000 ms	4000 ms	4000 ms	mean reaction time<br>game score<br>point rate
	complexity of the gear wheels (out of 3 levels)		Level 1	Level 1	Level 1	Level 1&2	Level 1&2	Level 1&2	Level 2	Level 2	Level 2&3	Level 3	
	animated (rotating wheels) wheels vs. wheels in fixed position		fixed	fixed	fixed	fixed	animated	animated	animated	animated	animated	animated	
	predictability (order/time interval)		random	random	random	random	random	random	random	random	random	random	
	stepping direction(s)	↑	80 %	60 %	40 %	35 %	35 %	30 %	30 %	25 %	25 %	25 %	
		→	10 %	20 %	25 %	25 %	25 %	25 %	25 %	25 %	25 %	25 %	
		←	10 %	20 %	25 %	25 %	25 %	25 %	25 %	25 %	25 %	25 %	
		↓	0 %	0 %	10 %	15 %	15 %	20 %	20 %	25 %	25 %	25 %	

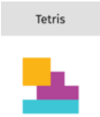	game speed (speed multiplier)	0.6	0.8	1	1.2	1.3	1.4	1.5	1.6	1.7	adaptive; start level: 1.8	game score

3 References

1. Boutron I, Altman DG, Moher D, Schulz KF, Ravaud P. CONSORT Statement for Randomized Trials of Nonpharmacologic Treatments: A 2017 Update and a CONSORT Extension for Nonpharmacologic Trial Abstracts. Annals of internal medicine. 2017;167(1):40-7. doi: https://doi.org/10.7326/M17-0046.
2. Manser P, de Bruin ED. Making the Best out of IT: Design and Development of Exergames for Older Adults with mild Neurocognitive Disorder – A Methodological Paper. In: Berry G, editor. Research Topic: Technological Advancements in Aging and Neurological Conditions to Improve Physical Activity, Cognitive Functions, and Postural Control. Frontiers in Aging Neuroscience2021.
3. Skivington K, Matthews L, Simpson SA, Craig P, Baird J, Blazeby JM, et al. A new framework for developing and evaluating complex interventions: update of Medical Research Council guidance. BMJ (Clinical research ed). 2021;374:n2061. doi: https://doi.org/10.1136/bmj.n2061.
4. Li Y, Muñoz J, Mehrabi S, Middleton L, Cao S, Boger J. Multidisciplinary Iterative Design of Exergames (MIDE): A Framework for Supporting the Design, Development, and Evaluation of Exergames for Health. International Conference on Human-Computer Interaction: Springer; 2020. p. 128-47.
5. Manser P, de Bruin ED. Making the Best Out of IT: Design and Development of Exergames for Older Adults With Mild Neurocognitive Disorder - A Methodological Paper. Front Aging Neurosci. 2021;13:734012. doi: https://doi.org/10.3389/fnagi.2021.734012.
6. Manser P, Adcock-Omlin M, de Bruin ED. Design Considerations for an Exergame-Based Training Intervention for Older Adults With Mild Neurocognitive Disorder: Qualitative Study Including Focus Groups With Experts and Health Care Professionals and Individual Semistructured In-depth Patient Interviews. JMIR Serious Games. 2023;11:e37616. doi: https://doi.org/10.2196/37616.
7. Manser P, Poikonen H, de Bruin ED. Feasibility, usability, and acceptance of “Brain-IT”—A newly developed exergame-based training concept for the secondary prevention of mild neurocognitive disorder: a pilot randomized controlled trial. Frontiers in Aging Neuroscience. 2023;15. doi: https://doi.org/10.3389/fnagi.2023.1163388.
8. Manser P, Michels L, Schmidt A, Barinka F, de Bruin ED. Effectiveness of an Individualized Exergame-Based Motor-Cognitive Training Concept Targeted to Improve Cognitive Functioning in Older Adults With Mild Neurocognitive Disorder: Study Protocol for a Randomized Controlled Trial. JMIR Resarch Protocols. 2023;12:e41173. doi: https://doi.org/10.2196/41173.
9. Slade SC, Dionne CE, Underwood M, Buchbinder R. Consensus on Exercise Reporting Template (CERT): Explanation and Elaboration Statement. British Journal of Sports Medicine. 2016;50(23):1428-37. doi: https://doi.org/10.1136/bjsports-2016-096651.
10. Herold F, Hamacher D, Schega L, Muller NG. Thinking While Moving or Moving While Thinking - Concepts of Motor-Cognitive Training for Cognitive Performance Enhancement. Frontiers in Aging Neuroscience. 2018;10(228). doi: https://doi.org/10.3389/fnagi.2018.00228.

11. Fissler P, Kuster O, Schlee W, Kolassa IT. Novelty interventions to enhance broad cognitive abilities and prevent dementia: synergistic approaches for the facilitation of positive plastic change. Prog Brain Res. 2013;207:403-34. doi: https://doi.org/10.1016/B978-0-444-63327-9.00017-5.

12. Bamidis PD, Vivas AB, Styliadis C, Frantzidis C, Klados M, Schlee W, et al. A review of physical and cognitive interventions in aging. Neurosci Biobehav Rev. 2014;44:206-20. doi: https://doi.org/10.1016/j.neubiorev.2014.03.019.

13. Garber CE, Blissmer B, Deschenes MR, Franklin BA, Lamonte MJ, Lee IM, et al. American College of Sports Medicine position stand. Quantity and quality of exercise for developing and maintaining cardiorespiratory, musculoskeletal, and neuromotor fitness in apparently healthy adults: guidance for prescribing exercise. Medicine and science in sports and exercise. 2011;43(7):1334-59. doi: https://doi.org/10.1249/MSS.0b013e318213fefb.

14. Ismail Z, Elbayoumi H, Fischer CE, Hogan DB, Millikin CP, Schweizer T, et al. Prevalence of Depression in Patients With Mild Cognitive Impairment: A Systematic Review and Meta-analysis. JAMA Psychiatry. 2017;74(1):58-67. doi: https://doi.org/10.1001/jamapsychiatry.2016.3162.

15. Ma LN. Depression, Anxiety, and Apathy in Mild Cognitive Impairment: Current Perspectives. Frontiers in Aging Neuroscience. 2020;12(9). doi: https://doi.org/10.3389/fnagi.2020.00009.

16. Shaffer F, McCraty R, Zerr CL. A healthy heart is not a metronome: an integrative review of the heart's anatomy and heart rate variability. Front Psychol. 2014;5:1040. doi: https://doi.org/10.3389/fpsyg.2014.01040.

17. Lehrer PM, Gevirtz R. Heart rate variability biofeedback: how and why does it work? Front Psychol. 2014;5:756. doi: https://doi.org/10.3389/fpsyg.2014.00756.

18. Zaccaro A, Piarulli A, Laurino M, Garbella E, Menicucci D, Neri B, et al. How Breath-Control Can Change Your Life: A Systematic Review on Psycho-Physiological Correlates of Slow Breathing. Front Hum Neurosci. 2018;12:353. doi: https://doi.org/10.3389/fnhum.2018.00353.

19. Schwerdtfeger AR, Schwarz G, Pfurtscheller K, Thayer JF, Jarczok MN, Pfurtscheller G. Heart rate variability (HRV): From brain death to resonance breathing at 6 breaths per minute. Clin Neurophysiol. 2020;131(3):676-93. doi: https://doi.org/10.1016/j.clinph.2019.11.013.

20. Laborde S, Allen MS, Borges U, Dosseville F, Hosang TJ, Iskra M, et al. Effects of voluntary slow breathing on heart rate and heart rate variability: A systematic review and a meta-analysis. Neuroscience & Biobehavioral Reviews. 2022;138:104711. doi: https://doi.org/10.1016/j.neubiorev.2022.104711.

21. Tinello D, Kliegel M, Zuber S. Does Heart Rate Variability Biofeedback Enhance Executive Functions Across the Lifespan? A Systematic Review. J Cogn Enhanc. 2022;6(1):126-42. doi: https://doi.org/10.1007/s41465-021-00218-3.

22. Lehrer P, Kaur K, Sharma A, Shah K, Huseby R, Bhavsar J, et al. Heart Rate Variability Biofeedback Improves Emotional and Physical Health and Performance: A Systematic

Review and Meta Analysis. Appl Psychophysiol Biofeedback. 2020;45(3):109-29. doi: https://doi.org/10.1007/s10484-020-09466-z.

23. Fincham GW, Strauss C, Montero-Marin J, Cavanagh K. Effect of breathwork on stress and mental health: A meta-analysis of randomised-controlled trials. Scientific Reports. 2023;13(1):432. doi: https://doi.org/10.1038/s41598-022-27247-y.

24. Goessl VC, Curtiss JE, Hofmann SG. The effect of heart rate variability biofeedback training on stress and anxiety: a meta-analysis. Psychol Med. 2017;47(15):2578-86. doi: https://doi.org/10.1017/S0033291717001003.

25. Jester DJ, Rozek EK, McKelley RA. Heart rate variability biofeedback: implications for cognitive and psychiatric effects in older adults. Aging Ment Health. 2019;23(5):574-80. doi: https://doi.org/10.1080/13607863.2018.1432031.

26. Min J, Rouanet J, Martini AC, Nashiro K, Yoo HJ, Porat S, et al. Modulating heart rate oscillation affects plasma amyloid beta and tau levels in younger and older adults. Scientific Reports. 2023;13(1):3967. doi: https://doi.org/10.1038/s41598-023-30167-0.

27. Laborde S, Ackermann S, Borges U, D'Agostini M, Giraudier M, Iskra M, et al. Leveraging Vagally Mediated Heart Rate Variability as an Actionable, Noninvasive Biomarker for Self-Regulation: Assessment, Intervention, and Evaluation. Policy Insights from the Behavioral and Brain Sciences. 2023;10(2):212-20. doi: https://doi.org/10.1177/23727322231196789.

28. Lehrer P, Vaschillo B, Zucker T, Graves J, Katsamanis M, Aviles M, et al. Protocol for Heart Rate Variability Biofeedback Training. Biofeedback. 2013;41(3):98-109. doi: https://doi.org/10.5298/1081-5937-41.3.08.

29. Karvonen MJ, Kentala E, Mustala O. The effects of training on heart rate; a longitudinal study. Ann Med Exp Biol Fenn. 1957;35(3):307-15.

30. Karvonen J, Vuorimaa T. Heart rate and exercise intensity during sports activities. Practical application. Sports medicine (Auckland, NZ). 1988;5(5):303-11. doi: https://doi.org/10.2165/00007256-198805050-00002.

31. Gentile AM. Skill Acquisition: Action, Movement, and Neuromotor Processes. In JH Carr R B Shepherd (Eds), Movement Science, Foundations for Physical Therapy in Rehabilitation. 2000;2nd Edition:111–87.

32. Kleim JA, Jones TA. Principles of experience-dependent neural plasticity: implications for rehabilitation after brain damage. J Speech Lang Hear Res. 2008;51(1):S225-39. doi: https://doi.org/10.1044/1092-4388(2008/018).

33. Maier M, Ballester BR, Verschure P. Principles of Neurorehabilitation After Stroke Based on Motor Learning and Brain Plasticity Mechanisms. Front Syst Neurosci. 2019;13:74. doi: https://doi.org/10.3389/fnsys.2019.00074.

34. Bayles MP. ACSM's exercise testing and prescription. Lippincott Williams & Wilkins; 2023.

35. Herold F, Müller P, Gronwald T, Müller NGJFip. Dose–Response Matters!–A Perspective on the Exercise Prescription in Exercise–Cognition Research. 2019;10.

36. Di Lorito C, Pollock K, Harwood R, das Nair R, Logan P, Goldberg S, et al. A scoping review of behaviour change theories in adults without dementia to adapt and develop

the 'PHYT in dementia', a model promoting physical activity in people with dementia. Maturitas. 2019;121:101-13. doi: https://doi.org/10.1016/j.maturitas.2019.01.008.

37. Wulf G, Lewthwaite R. Optimizing performance through intrinsic motivation and attention for learning: The OPTIMAL theory of motor learning. Psychon Bull Rev. 2016;23(5):1382-414. doi: https://doi.org/10.3758/s13423-015-0999-9.

38. Lemos A, Wulf G, Lewthwaite R, Chiviacowsky S. Autonomy support enhances performance expectancies, positive affect, and motor learning. Psychology of Sport and Exercise. 2017;31:28-34. doi: https://doi.org/10.1016/j.psychsport.2017.03.009.

39. Giannouli E, Morat T, Zijlstra W. A Novel Square-Stepping Exercise Program for Older Adults (StepIt): Rationale and Implications for Falls Prevention. Front Med (Lausanne). 2019;6:318. doi: https://doi.org/10.3389/fmed.2019.00318.
