# Supplementary material for: “Brain‐IT”: Exergame training with biofeedback breathing in neurocognitive disorders
Source: Alzheimers Dement. 2024 May 29;20(7):4747–64. doi: 10.1002/alz.13913 (PMC11247687; doi:10.1002/alz.13913)
Supplement: Supplementary file 2 — Supporting Information [file ALZ-20-4747-s001.pdf]

ICMJE DISCLOSURE FORM

Date: 4/10/2023

Your Name: Patrick Manser

Manuscript Title: ‘Brain-IT’ - exergame training with biofeedback breathing in neurocognitive disorders

Manuscript Number (if known): ADJ-D-24-00341

In the interest of transparency, we ask you to disclose all relationships/activities/interests listed below that are related to the content of your manuscript. “Related” means any relation with for-profit or not-for-profit third parties whose interests may be affected by the content of the manuscript. Disclosure represents a commitment to transparency and does not necessarily indicate a bias. If you are in doubt about whether to list a relationship/activity/interest, it is preferable that you do so.

The author’s relationships/activities/interests should be defined broadly. For example, if your manuscript pertains to the epidemiology of hypertension, you should declare all relationships with manufacturers of antihypertensive medication, even if that medication is not mentioned in the manuscript.

In item #1 below, report all support for the work reported in this manuscript without time limit. For all other items, the time frame for disclosure is the past 36 months.

		Name all entities with whom you have this relationship or indicate none (add rows as needed)	Specifications/Comments (e.g., if payments were made to you or to your institution)
**Time frame: Since the initial planning of the work**			
1	All support for the present manuscript (e.g., funding, provision of study materials, medical writing, article processing charges, etc.) **No time limit for this item.**	☐ **None**	
		Project-specific funding: Synapsis Foundation-Dementia Research Switzerland (research grant 2019-PI06) and the “Gebauer Stiftung”	Project-specific funding was paid to the institution of employment, ETH Zurich, yearly from 2021 to 2024.
		Financial Support: “Fondation Dalle Molle”	Prize money for the “Quality of life” label was paid to the institution of employment, ETH Zurich, in 2021.
		Providing of technology	8 “Senso Flex” training systems were provided free of charge by Dividat AG for the duration of the study.
		APC funding	APC charges were covered by the ETH Zurich under an agreement with Wiley.
		Comment: None of the above listed funders nor Dividat AG played any role in the design of this study, nor did they play any role in the collection, management, analysis, and interpretation of data, writing of the report, or decision to submit the report for publication.	
**Time frame: past 36 months**			
2	Grants or contracts from any entity (if not indicated in item #1 above).	☒ **None**	

		Name all entities with whom you have this relationship or indicate none (add rows as needed)	Specifications/Comments (e.g., if payments were made to you or to your institution)
**3**	Royalties or licenses	☒ **None**	
**4**	Consulting fees	☒ **None**	
**5**	Payment or honoraria for lectures, presentations, speakers bureaus, manuscript writing or educational events	☒ **None**	
**6**	Payment for expert testimony	☒ **None**	
**7**	Support for attending meetings and/or travel	☒ **None**	
**8**	Patents planned, issued or pending	☒ **None**	
**9**	Participation on a Data Safety Monitoring Board or Advisory Board	☒ **None**	
**10**	Leadership or fiduciary role in other board,	☒ **None**	

		Name all entities with whom you have this relationship or indicate none (add rows as needed)	Specifications/Comments (e.g., if payments were made to you or to your institution)
	society, committee or advocacy group, paid or unpaid		
**11**	Stock or stock options	☒ **None**	
**12**	Receipt of equipment, materials, drugs, medical writing, gifts or other services	☐ **None**<br>Providing of technology	"Senso Flex" training systems were provided free of charge by Dividat AG for the duration of the study.
		Comment: Dividat AG played no role in the design of this study, nor did they play any role in the collection, management, analysis, and interpretation of data, writing of the report, or decision to submit the report for publication.	
**13**	Other financial or non-financial interests	☒ **None**	

Please place an "X" next to the following statement to indicate your agreement:

☒ I certify that I have answered every question and have not altered the wording of any of the questions on this form.

ICMJE DISCLOSURE FORM

**Date:**	11/4/2023
**Your Name:**	Prof. Dr. Eling D. de Bruin
**Manuscript Title:**	‘Brain-IT’ - exergame training with biofeedback breathing in neurocognitive disorders
**Manuscript Number (if known):**	ADJ-D-24-00341

In the interest of transparency, we ask you to disclose all relationships/activities/interests listed below that are related to the content of your manuscript. “Related” means any relation with for-profit or not-for-profit third parties whose interests may be affected by the content of the manuscript. Disclosure represents a commitment to transparency and does not necessarily indicate a bias. If you are in doubt about whether to list a relationship/activity/interest, it is preferable that you do so.

The author’s relationships/activities/interests should be defined broadly. For example, if your manuscript pertains to the epidemiology of hypertension, you should declare all relationships with manufacturers of antihypertensive medication, even if that medication is not mentioned in the manuscript.

In item #1 below, report all support for the work reported in this manuscript without time limit. For all other items, the time frame for disclosure is the past 36 months.

		**Name all entities with whom you have this relationship or indicate none (add rows as needed)**	**Specifications/Comments (e.g., if payments were made to you or to your institution)**
**Time frame: Since the initial planning of the work**			
**1**	All support for the present manuscript (e.g., funding, provision of study materials, medical writing, article processing charges, etc.) **No time limit for this item.**	☐ **None**	
		Project-specific funding: Synapsis Foundation-Dementia Research Switzerland (research grant 2019-PI06) and the “Gebauer Stiftung”	Project-specific funding was paid to the institution of employment, ETH Zurich, yearly from 2021 to 2024.
		Financial Support: “Fondation Dalle Molle”	Prize money for the “Quality of life” label was paid to the institution of employment, ETH Zurich, in 2021.
		Providing of technology	8 “Senso Flex” training systems were provided free of charge by Dividat AG for the duration of the study.
		APC funding	APC charges were covered by the ETH Zurich under an agreement with Wiley.
		Comment: None of the above listed funders nor Dividat AG played any role in the design of this study, nor did they play any role in the collection, management, analysis, and interpretation of data, writing of the report, or decision to submit the report for publication.	
**Time frame: past 36 months**			
**2**	Grants or contracts from any entity (if not indicated in item #1 above).	☒ **None**	

		Name all entities with whom you have this relationship or indicate none (add rows as needed)	Specifications/Comments (e.g., if payments were made to you or to your institution)
**3**	Royalties or licenses	☒ **None**	
**4**	Consulting fees	☒ **None**	
**5**	Payment or honoraria for lectures, presentations, speakers bureaus, manuscript writing or educational events	☒ **None**	
**6**	Payment for expert testimony	☒ **None**	
**7**	Support for attending meetings and/or travel	☒ **None**	
**8**	Patents planned, issued or pending	☒ **None**	
**9**	Participation on a Data Safety Monitoring Board or Advisory Board	☒ **None**	
**10**	Leadership or fiduciary role in other board,	☒ **None**	

		Name all entities with whom you have this relationship or indicate none (add rows as needed)	Specifications/Comments (e.g., if payments were made to you or to your institution)
	society, committee or advocacy group, paid or unpaid		
**11**	Stock or stock options	☒ **None**	
**12**	Receipt of equipment, materials, drugs, medical writing, gifts or other services	☐ **None**	
		Providing of technology	"Senso Flex" training systems were provided free of charge by Dividat AG for the duration of the study.
		Comment: Dividat AG played no role in the design of this study, nor did they play any role in the collection, management, analysis, and interpretation of data, writing of the report, or decision to submit the report for publication.	
**13**	Other financial or non-financial interests	☒ **None**	

Please place an "X" next to the following statement to indicate your agreement:

☒ I certify that I have answered every question and have not altered the wording of any of the questions on this form.
